# Catalytic Supercritical Water Gasification of Canola Straw with Promoted and Supported Nickel-Based Catalysts

**DOI:** 10.3390/molecules29040911

**Published:** 2024-02-19

**Authors:** Kapil Khandelwal, Ajay K. Dalai

**Affiliations:** Catalysis and Chemical Reaction Engineering Laboratories, Department of Chemical and Biological Engineering, University of Saskatchewan, Saskatoon, SK S7N 5A9, Canada; kak368@usask.ca

**Keywords:** canola straw, hydrogen, nickel catalysts, catalytic gasification, supercritical water gasification

## Abstract

Lignocellulosic biomass such as canola straw is produced as low-value residue from the canola processing industry. Its high cellulose and hemicellulose content makes it a suitable candidate for the production of hydrogen via supercritical water gasification. However, supercritical water gasification of lignocellulosic biomass such as canola straw suffers from low hydrogen yield, hydrogen selectivity, and conversion efficiencies. Cost-effective and sustainable catalysts with high catalytic activity for supercritical water gasification are increasingly becoming a focal point of interest. In this research study, novel wet-impregnated nickel-based catalysts supported on carbon-negative hydrochar obtained from hydrothermal liquefaction (HTL-HC) and hydrothermal carbonization (HTC-HC) of canola straw, along with other nickel-supported catalysts such as Ni/Al_2_O_3_, Ni/ZrO_2_, Ni/CNT, and Ni/AC, were synthesized for gasification of canola straw on previously optimized reaction conditions of 500 °C, 60 min, 10 wt%, and 23–25 MPa. The order of hydrogen yield for the six supports was (10.5 mmol/g) Ni/ZrO_2_ > (9.9 mmol/g) Ni/Al_2_O_3_ > (9.1 mmol/g) Ni/HTL-HC > (8.8 mmol/g) Ni/HTC-HC > (7.7 mmol/g) Ni/AC > (6.8 mmol/g) Ni/CNT, compared to 8.1 mmol/g for the non-catalytic run. The most suitable Ni/ZrO_2_ catalyst was further modified using promotors such as K, Zn, and Ce, and the performance of the promoted Ni/ZrO_2_ catalysts was evaluated. Ni-Ce/ZrO_2_ showed the highest hydrogen yield of 12.9 mmol/g, followed by 12.0 mmol/g for Ni-Zn/ZrO_2_ and 11.6 mmol/g for Ni-K/ZrO_2_. The most suitable Ni-Ce/ZrO_2_ catalysts also demonstrated high stability over their repeated use. The superior performance of the Ni-Ce/ZrO_2_ was due to its high nickel dispersion, resilience to sintering, high thermal stability, and oxygen storage capabilities to minimize coke deposition.

## 1. Introduction

Global energy consumption is ever-increasing due to rapid globalization and urbanization. In 2022, global energy consumption had reached a staggering 630 Exajoules (EJ), which is a 1.11% increase from the 2021 level [1,2]. Nearly 81% of the global energy demand is fulfilled by non-renewable fossil fuel sources, namely natural gas, oil, and coal. The incineration of these fossil fuels for the production of energy emits large amounts of greenhouse gases (GHGs), resulting in various environmental challenges, namely climate change, floods, weather pattern changes, and unpredictable monsoon cycles. United Nations (UN) experts have called the human-induced climate change the most pervasive threat to human existence [3], needing a clean and sustainable source of energy production to prevent it.

Hydrogen-based energy sources have gained attention as a clean source of energy due to their combustion products of water and energy [4]. Demand for hydrogen is also increasing in industries and refineries for a variety of processes, such as ammonia and methanol production, hydrodeoxygenation, and hydrodesulfurization processes [5,6,7]. In 2022, global hydrogen demand reached 95 Mt, a 3% increase from the 2021 global hydrogen demand [8]. Despite the clean nature of hydrogen, it is primarily produced by non-renewable fossil fuel sources and its byproducts. Less than 1 Mt of hydrogen, which is 0.7% of global hydrogen production, is produced via low-emission sources [8]. To fully realize the benefits of the hydrogen economy, hydrogen should be produced via green and sustainable sources.

Lignocellulosic biomass is a great source of energy and production of biofuels [9]. Lignocellulosic biomass resources mostly comprise agriculture residues, wood chips, forestry residues, and biogenic waste materials. These biomass resources are cost-effective and abundantly available. Canola production is on the rise in Canada, and in 2022, out of 31 million metric tons of canola produced worldwide, 20 million metric tons of canola crop were produced in Canada. Annual production of canola in Canada is further expected to grow to 26 million tons by 2026 [10]. Canola straw is a leftover byproduct of the canola crop after the removal of grains and chaff from the crop and accounts for nearly 55% of the total crop. Canola straw is currently used as bedding material for livestock. However, most of the canola straw is unutilized and has very low value. These canola straws are rich in cellulose and hemicellulose content, which can be easily converted into simpler sugar molecules and subsequently into biofuels.

Unlike fossil fuels, these biomass sources contain and have the tendency to absorb large amounts of moisture, which leads to decrease in the biomass stiffness and its structural strength [11,12]. This requires pre-drying of the feedstock in the conventional thermochemical process, which increases the heat duty and makes the process energy-inefficient. Hydrothermal gasification of biomass in supercritical water (SCW) overcomes this limitation for the production of H_2_ without needing to pre-dry the feedstock [13]. Supercritical water gasification of biomass (SCWG) is conducted above the critical point of water, which exists at temperatures greater than 374 °C and pressure larger than 22.1 MPa [14]. Supercritical water exhibits unique properties such as non-polar nature, high diffusivity, mono-phase reaction mechanism, and high solubility of gases, which imparts low mass transfer limitation, easy product separation, and high thermal efficiency to the SCWG process [15].

In our previous study, canola straw was gasified in the presence of supercritical water, and the effects of SCWG reaction conditions on the gas yield and mechanism of the gasification were studied [16]. An increase in reaction time and reaction temperature generally improved the hydrogen yield, whereas a rise in feedstock concentration minimized the hydrogen production. The highest H_2_ yield achieved was 8.1 mmol/g at the optimized conditions of 500 °C, 23–25 MPa, 10 wt%, and 60 min. Despite the successful gasification of canola straw in supercritical water, it had its own set of challenges. A primary issue is the relatively low yield of the gaseous products, resulting in low conversion efficiencies. This results in ineffective utilization of valuable biomass resources, which remained underutilized. Furthermore, gaseous products also showed relatively lower selectivity for hydrogen gas. This is due to the slow kinetics of reforming reactions and water–gas shift reactions, which are responsible for the gasification of biomass into hydrogen-rich gaseous products. These reactions have high activation energies which require catalysts for rapid reaction rates, explaining low gas yields and hydrogen selectivity in non-catalytic gasification. Usually, homogenous alkali metal salt catalysts, namely potassium carbonate (K_2_CO_3_), sodium hydroxide (NaOH), potassium hydroxide (KOH), and sodium carbonate (Na_2_CO_3_), are utilized for SCWG of biomass [17,18,19]. Alkali metal salts promote a water–gas shift (WGS) reaction by forming a formate intermediate compound to shift the equilibrium towards the product side and enhance hydrogen yield. However, recovery and reusability of the homogenous alkali metals are challenging [20]. 

Heterogenous noble metal catalysts such as nickel are widely used catalysts in SCWG due to their cost effectiveness, enhancement of the WGS reaction, and comparable activity to more expensive noble metal catalysts [21]. Due to their superior catalytic activity, researchers have utilized novel modified nickel catalysts with various supports and promotors. Lu et al. [22] compared nickel (Ni), iron (Fe), and copper (Cu) metal catalysts supported on magnesium oxide (MgO) for SCWG of wheat stalk. The results show that the Ni/MgO demonstrated high catalytic performance, with the highest hydrogen yield of 11.6 mmol/g. Similarly, Su et al. [23] synthesized a novel lanthanum (La)-promoted nickel (Ni) catalyst supported on alumina (Al_2_O_3_) for the gasification of food waste in supercritical water. This catalyst nearly doubled the hydrogen and total gas yield compared to that in a non-catalytic run.

Development of cost-effective and green catalysts from low-value waste streams of other biorefinery processes in integrated matter for supercritical water gasification is gaining popularity [24]. This will help us to not only efficiently utilize the biomass resources from other biorefinery processes but also to contribute to the circular bioeconomy for cost-effective production of biofuels. Hydrothermal liquefaction and hydrothermal carbonization of biomass produce valuable products such as bio-oil, bio-gas, and aqueous phase. These processes also produce low-value carbon-negative hydrochar which has a high surface area [25]. These hydrochars are rich in alkali and alkaline earth metals (AAEMs). These AAEMs have catalytic effects in supercritical water gasification by enhancing reforming and water–gas shift reactions to maximize hydrogen yield. Despite its great potential for use as a catalyst support in SCWG, no study is available on the use of hydrochar as a catalyst support for the SCWG of biomass.

Carbonaceous supports such as activated carbon (AC) and carbon nanotube (CNT) are biodegradable and renewable in nature with a high surface area, making them suitable support for SCWG catalysts [26,27]. Similarly, alumina (Al_2_O_3_) and zirconia (ZrO_2_) are also widely used Ni catalyst supports for SCWG due to their ability to promote reforming and WGS reactions [28,29,30]. Even though studies are available for the utilization of these Ni-supported catalysts for the SCWG of model compounds, only a few studies are available for the utilization of these catalysts for the SCWG of lignocellulosic biomass.

Furthermore, these studies have also reported that Ni catalysts suffer from sintering and enhancement of side reactions, such as methanation reactions, which consume the produced hydrogen. In the reaction conditions of SCWG, Ni catalysts also suffer from coking and sintering. The stability and resilience of catalysts are critical parameters for the selection of suitable catalysts for the SCWG. To overcome these challenges, researchers have employed various promotors that can be used for the enhancement of the stability and activity of the SCWG catalyst. Furthermore, few literature studies are available on the screening and comparison of various promotors for nickel-based catalysts for the gasification of lignocellulosic biomass.

To address these knowledge gaps, in this study, we have synthesized and compared wet-impregnated novel nickel-supported catalysts on hydrochar obtained from hydrothermal liquefaction (HTL-HC) and hydrothermal carbonization (HTC-HC), alumina (Al_2_O_3_), zirconia (ZrO_2_), activated carbon (AC), and carbon nanotube (CNT) for supercritical water gasification of canola straw. The most suitable supported nickel catalyst was further modified with the addition of promotors. Three promotors, potassium (K), zinc (Zn), and cerium (Ce), were systematically screened for the supported nickel catalysts. An in-depth investigation of the activity, stability, textural, and physical properties of synthesized catalysts was conducted. Furthermore, the reaction mechanism of the catalytic action of nickel-based catalysts was developed to study the relationship between the characteristics of the catalysts and the degradation of canola straw in supercritical water gasification.

## 2. Materials and Methods

### 2.1. Preparation of Feedstock and Catalysts

Canola straw was utilized as the feedstock for the supercritical water gasification experiments. It was sourced from a local Canadian farmer in Saskatchewan. Canola straw was dried and pulverized to a uniform particle size of 1 mm. Elemental analysis (C = 46.3%, H = 6.8%, N = 0.9%, S = 0.4%, and O = 45.6%) and compositional analysis (cellulose = 46.2%, hemicellulose = 29.2%, and lignin = 14.2%) of canola straw have been reported in our previous study [16]. Metal precursors utilized for synthesis of catalysts nickel (II) nitrate hexahydrate [Ni(NO_3_)_2_·6H_2_O], cerium (III) nitrate hexahydrate [Ce(NO_3_)_3_·6H_2_O], zinc (II) nitrate hexahydrate [Zn(NO_3_)_3_·6H_2_O], and potassium nitrate (KNO_3_) were procured from Thermo Fisher Scientific, Mississauga, ON, Canada. Catalyst support Al_2_O_3_ was purchased from Thermo Fisher Scientific, Mississauga, ON, Canada, and ZrO_2_ was purchased from Alfa Aesar, Ward Hill, MA, USA. AC was purchased from Calgon Carbon, Caledon, ON, Canada, and CNT was purchased from M K Impex Corp., Mississauga, ON, Canada, both of which were subsequently functionalized by using acid reflux with a mixture of HNO_3_ and H_2_SO_4_ at a temperature of 90 °C for 5 h.

Hydrochar from hydrothermal carbonization was synthesized by performing HTC of the canola straw in a stainless-steel batch reactor with a capacity of 1.8 L. The HTC reaction was performed at the reaction conditions of 260 °C, 60 min, and feed-to-water ratio of 1:5. Upon completion of the HTC reaction, reaction products were filtered through a vacuum filtration using a cellulose filter paper of 15–19 μm grade. HTC-hydrochar was recovered from the top of the vacuum filter and dried at 105 °C in an oven for 12 h. Similarly, hydrothermal liquefaction of canola straw was performed in a stainless-steel Parr batch reactor with a capacity of 900 mL at reaction conditions of 300 °C, 60 min, and feed-to-water ratio of 1:5. HTL reaction products were separated using vacuums filtration with a cellulose filter paper to sperate aqueous phase from mixture of bio-oil and hydrochar. HTL hydrochar was separated from bio-oil using solvent extraction followed by a series of vacuum filtrations. Recovered HTL-hydrochar was then dried at 105 °C in an oven for 12 h.

All the catalysts used in this study were synthesized via the incipient wetness impregnation method, and promoted catalysts were prepared using co-impregnation. The optimized amount of metal precursors for metal loading was considered from the literature on the optimization of nickel-based catalysts for SCWG [31,32,33]. Most of the studies have considered the optimal effects of nickel metal to be a loading of 10 wt% for the maximum performance of nickel-based catalysts in SCWG. This is due to the enhancement in gas yield with increase in nickel metal loading; however, at higher metal loading, catalyst particles suffer from agglomeration. Therefore, in this study, an optimized metal precursor loading of 10 wt% was considered, and the amounts of nickel and promotor metal precursors were calculated based on the designed composition of the catalyst. First, distilled water was used to dissolve metal precursors, and then, using a 1 mL syringe, this mixture was impregnated on the support. Catalysts were then left for 2 h for aging to attain equilibrium and thereafter dried at 105 °C in an oven for 12 h. Catalysts supported on metal oxide supports (Al_2_O_3_ and ZrO_2_) were calcined under air, and catalysts supported on carbon supports such as CNT, AC, HTL-HC, and HTC-HC were calcined under an inert environment in the presence of nitrogen at a temperature of 650 °C for 4 h. An inert environment was maintained to avoid the oxidation of carbon supports.

### 2.2. Experimental Procedure for SCWG Reaction

SCWG reactions were performed in a batch reactor made up of stainless steel 316 with a 40 cm length, 1.3 cm outer diameter, and 0.9 cm internal diameter. The schematics and workings of the SCWG reactor assembly were discussed in detail in our previous publication [16]. The reactor assembly consisted of a K-type thermocouple, an ATS split furnace with a temperature controller system for heating, check valves, a pressure gauge, a pressure relief valve, a gas–liquid separator, 2 μm filters, and a dehydrating desiccant column. For each experimental run, 1.1 g of canola straw was mixed with the appropriate amount of deionized water to make a feedstock mixture and fed to the reactor along with 1 gm of desired catalysts for the catalytic runs. All experiments were conducted at the previously optimized SCWG reaction conditions of 500 °C, 23–25 MPa, 10 wt%, and 60 min. Nitrogen gas was used for purging and establishment of initial pressure of 8–10 MPa. After the completion of the SCWG reaction at the desired reaction time of 60 min, reaction products were sent to gas–liquid separators to recover liquid products, and gaseous products were sent to a dehydrating desiccant column (LabClear Drierite^®^) for removal of any trace amount of moisture. Moisture-free gaseous products were then collected in a tedlar bag for the gas chromatography (GC) analysis. Spent catalysts were collected along with biochar after sufficient cooling of the reactor using distilled water. The spent catalyst was then sequentially washed with tetrahydrofuran and distilled water for removal of any organics. Washed spent catalysts were then filtered and oven-dried at 60 °C for 12 h.

### 2.3. Characterization of Catalysts and SCWG Products

Wide-angle powder X-ray diffraction (XRD) of all catalysts was conducted using Cu Kα in a Bruker D8 ADVANCE diffractometer purchased from Bruker AXS, Karlsruhe, Germany. XRD analysis was conducted in a scanning range of 10–90°. Thermogravimetric analysis (TGA) of catalysts to determine the thermal stability of catalysts and resilience to coking was conducted in a Q500 TGA instrument purchased from TA Instruments-Waters, USA. TGA was conducted in an inert N_2_ environment from room temperature to 600 °C with a heating rate of 10 °C/min. Brunauer–Emmett–Teller (BET) analysis of fresh and spent catalysts was performed to evaluate the pore volume and surface area of the catalysts and effect of SCWG reactions on the structure of the catalyst in a Micrometrics ASAP 2020 instrument purchased from Micrometrics, Norcross, USA. Samples were first degassed at 90 °C for 1 h and 350 °C for 4 h under a pressure of 0.5 mm Hg, followed by nitrogen adsorption and desorption analyses in a Micrometrics ASAP 2020 instrument at −196 °C. Scanning electron microscopy–energy dispersive X-ray analysis (SEM-EDX) analysis of catalysts was conducted to analyze the morphology and distribution of elements in fresh and spent catalysts using the SU8010 SEM from Hitachi and the Ultim Max 170 EDX from Oxford Instruments. Samples were coated with chromium, and data were collected at 15 kV accelerating voltage. Elemental analyses of fresh and spent catalysts were also conducted using inductively coupled plasma optical emission spectroscopy (ICP-OES) analysis of catalysts with a Sciex Elan 5000 ICP-MS instrument purchased from PerkinElmer, Inc., Waltham, MA, USA.

The composition of gases from SCWG of canola residue was measured by an Agilent 7820A gas chromatography that had a capillary column with three mesh columns. Helium and nitrogen were used as the carrier gases for the analysis. The individual gas yield of *i*^th^ from gaseous products was estimated with Equation (1).

(1) $${Yield}_{i}\;(mmol/g)=\frac{Produced\;moles\;of\;i^{th}\;gas\;(mmol)}{Weight\;of\;feedstock\;(g)}$$

Hydrogen selectivity of the gaseous product was determined using Equation (2).

(2) $$H_{2}\;selectivity\;(\%)=\frac{Hydrogen\;yield\;(mmol)}{Total\;gas\;yield\;(mmol)}\times 100$$

The lower heating value (LHV) of gases was estimated using Equation (3) [31].

(3) $$LHV\;\left(\frac{kJ}{Nm^{3}}\right)=4.2\times (30.3\times CO+25.8\times H_{2}+85.5\times CH_{4}+151.3\times C_{n}H_{m})$$

In Equation (3), CO, H_2_, CH_4_, and C_n_H_m_ represent the mole fraction of each of these gases, and 30.3, 25.8, 85.5, and 151.3 are energy content values for CO, H_2_, CH_4_, and C_n_H_m_, respectively, in $\left(\frac{MJ}{Nm^{3}}\right)$. In addition, 4.2 is the constant for conversion of energy content from $\left(\frac{MJ}{Nm^{3}}\right)$ to $\left(\frac{kJ}{Nm^{3}}\right)$.

## 3. Results and Discussions

### 3.1. Characterization and Screening of Supports for Nickel-Based Catalysts

Nickel-based catalysts are efficient for degradation of biomass in SCWG. Nickel promotes the reforming and water–gas shift reactions to enhance hydrogen yield. However, using only nickel metal catalysts for SCWG resulted in a marginal improvement of 9.4% from 8.1 mmol/g of non-catalytic run. This is due to low availability of metal surface area due to low Ni dispersion, as the activity of catalysts is proportional to the available surface area of active metal. Therefore, catalyst supports are used to enhance the dispersion of nickel metal to significantly increase the active metal surface area, which results in improved performance of the catalyst for the same amount of active metal loading. Furthermore, catalyst support also plays a key role in the activity, stability, and durability of the metal catalysts, especially for the SCWG reaction, which employs severe reaction conditions. Therefore, in this study, six different supports, namely zirconium dioxide (ZrO_2_), carbon nanotubes (CNTs), activated carbon (AC), aluminum oxide (Al_2_O_3_), hydrothermal liquefaction-hydrochar (HTL-HC), and hydrothermal carbonization-hydrochar (HTC-HC), were synthesized for nickel-based catalysts. The physical properties and activity of these supported catalysts for SCWG were evaluated for the selection of the most suitable support for further modification.

**Figure 1:**
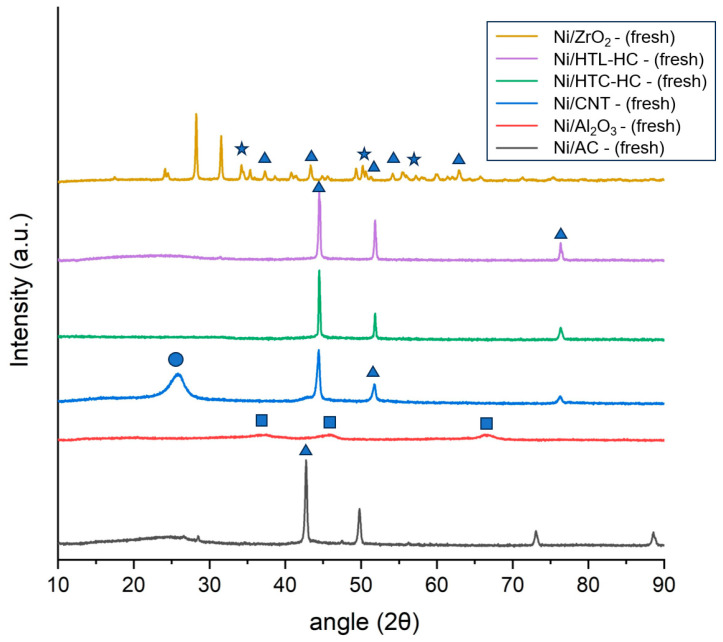
X-ray diffraction (XRD) analysis of fresh nickel catalysts supported on AC, CNT, HTC-HC, HTL-HC, Al_2_O_3_, and ZrO_2_ supports. NiO is represented as (▲), ZrO_2_ as (★), crystalline graphite as (●), and Al_2_O_3_ as (■) in the curve.

Crystallographic phases of fresh catalysts were studied using powdered X-ray diffraction analysis (XRD). Figure 1 represents the XRD pattern of the fresh Ni catalysts supported on AC, CNT, HTC-HC, HTL-HC, Al_2_O_3_, and ZrO_2_. It can be identified that nickel in all catalysts was present in the oxide form and identified by the peaks of NiO observed at the 2θ angle of 37°, 44°, 52°, 62.5°, 67°, and 76° [28,34]. Small shifts in catalysts can be assigned to the difference in the metal–support interactions of catalysts. For Ni/Al_2_O_3_, a broad peak was observed at the 2θ angle of 36°, 46°, and 67° in the XRD pattern, representing the crystalline phase of Al_2_O_3_. The XRD pattern of Ni/ZrO_2_ showed a distinct crystalline structure with its sharp peaks. Peaks observed at the 2θ angle of 28°, 31°, 34°, and 56° represent the (111), (111), (020), and (130) planes of monoclinic ZrO_2_. However, other peaks observed at the 2θ angle of 30°, 35°, 51°, and 60° represent the (101), (110), (200), and (211) planes of tetragonal ZrO_2_. This indicates that the ZrO_2_ present in the catalyst support was a mixture of both tetragonal and monoclinic ZrO_2_. However, it can be identified that the intensity of planes of monoclinic ZrO_2_ was higher than the intensity of planes of tetragonal ZrO_2_. This represents the fact that the proportion of the monoclinic phase of ZrO_2_ was higher in catalyst support than the tetragonal ZrO_2_. For CNT catalysts, a distinct peak observed at 2θ of 25° represents the crystalline carbon peak of the CNT. Among all the catalysts, the intensity of the NiO peak at 2θ of 44° was the minimum for ZrO_2_- and Al_2_O_3_-supported catalysts. This represents the higher dispersion of Ni on these catalyst supports, whereas the intensity of NiO peaks was highest in both Ni/AC and Ni/CNT catalysts. Additionally, in all carbon supports, a distinct peak of NiO at 2θ of 76° was visible as compared to other non-carbon supports. The intensity of this peak was highest in Ni/CNT, closely followed by Ni/AC. This corresponds to the larger particle size of nickel in both catalysts. This also explains the relatively superior performance of the ZrO_2_ catalyst and poor performance of Ni/CNT and Ni/AC.

**Figure 2:**
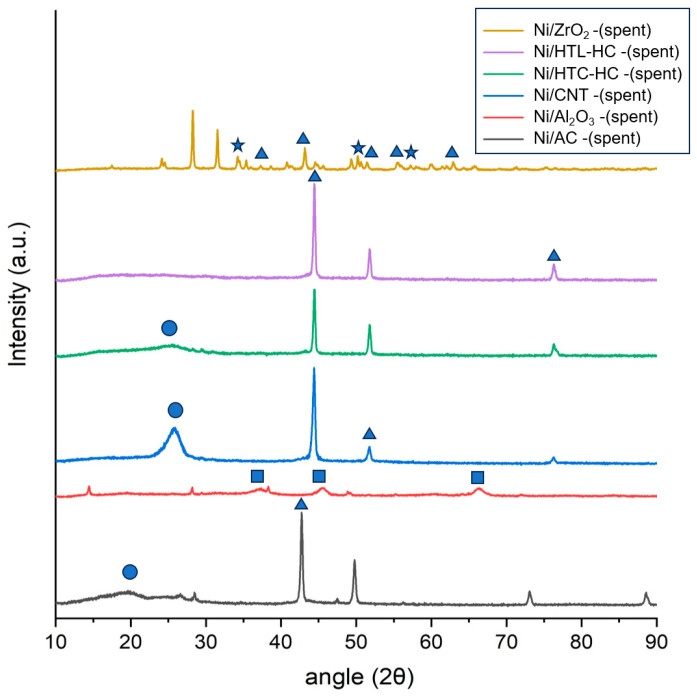
X-ray diffraction analysis (XRD) analysis of spent nickel catalysts supported on AC, CNT, HTC-HC, HTL-HC, Al_2_O_3_, and ZrO_2_ supports. NiO is represented as (▲), ZrO_2_ as (★), crystalline graphite as (●), and Al_2_O_3_ as (■) in the curve.

In XRD diffraction analysis of spent Ni-supported catalysts in SCWG of canola after they were used for gasification of canola straw in SCW, a broad peak in the region of 13–26° was observed (Figure 2). This peak was not identified in the fresh catalysts, and it represents the amorphous carbon possibly originating through coke formation in the spent catalyst. In all spent catalysts, a significant rise in NiO peaks was observed compared with those of the fresh catalyst. This shows that during the gasification of the canola straw in SCW, the catalyst suffers from the growth in the particle size of Ni particles. Among all spent catalysts, the intensity of the NiO peak for the Ni/CNT catalyst compared to the fresh catalyst increased quite significantly, followed by the Ni/AC catalyst. This represents the fact that the growth in Ni particles on the CNT surface was higher during SCWG than for other catalysts, which significantly reduced the activity of the catalysts in gasification. For the Ni/ZrO_2_ catalyst, however, the increase in the intensity of the NiO peak compared to the fresh catalyst was small. This shows that the Ni particles were still dispersed at the ZrO_2_ support surface and did not agglomerate, thus retaining higher catalytic activity. A probable reason for this behavior might be the difference in metal–support interaction in various nickel-supported catalysts, which can affect the growth of NiO particles during the gasification reaction in SCW.

Brunauer–Emmett–Teller (BET) analysis of fresh catalysts was conducted to evaluate the surface properties of supported nickel catalysts, and results are presented in Table 1. The BET surface area of 590 m^2^/g of Ni/AC was the highest, followed by 291 m^2^/g of Ni/CNT, 280 m^2^/g of Ni/Al_2_O_3_, 59 m^2^/g of Ni/HTL-HC, 45 m^2^/g of Ni/HTC-HC, and 6 m^2^/g of Ni/ZrO_2_. Meanwhile, the total pore volume of (0.93 cm^3^/g) Ni/CNT was the highest. This is due to the use of multi-walled CNT as a support in the synthesis of Ni/CNT. The order of total pore volume was (0.93 cm^3^/g) Ni/CNT > (0.68 cm^3^/g) Ni/AC > (0.58 cm^3^/g) Ni/Al_2_O_3_ > (0.07 cm^3^/g) Ni/HTL-HC > (0.03 cm^3^/g) Ni/HTC-HC > (0.02 m^2^/g) Ni/ZrO_2_. However, the pore size of (12.8 nm) Ni/ZrO_2_ was very high and comparable to the pore size of 17.1 nm of Ni/CNT. BET analysis of spent catalysts post SCWG of canola straw was also conducted to study the changes in surface properties of supported nickel catalysts during gasification in SCW.

From Table 1, it can also be identified that for all spent catalysts, the BET surface area decreased compared to the BET surface area of fresh catalysts. Similarly, the pore volume of spent catalysts was lower than that of the fresh counterpart. This can be explained by the fact that during gasification in SCW, pores of catalysts start to clog as the size of nickel crystals starts to grow in catalysts due to sintering, as indicated by an increase in the intensity of NiO peaks in XRD analysis of spent catalysts as compared to their fresh counterparts. This leads to lower pore volume and lower BET surface area of the spent catalysts. The order of the BET surface areas of spent catalysts followed a similar trend to the BET surface areas of fresh catalysts. The extent of drop in the BET surface area of spent Ni/AC catalysts as compared to fresh Ni/AC catalysts was very drastic and highest compared to other catalysts. This was due to severe coking in the Ni/AC catalyst which decreased the surface area of the spent catalyst. This was also accompanied by its drastic drop in pore volume. Change in the BET surface area and pore volume of spent catalysts compared to fresh catalysts was lowest in the case of Ni/ZrO_2_ catalysts. This highlights the excellent structural stability of the Ni/ZrO_2_ catalyst during the gasification of canola straw in SCW.

**Figure 3:**
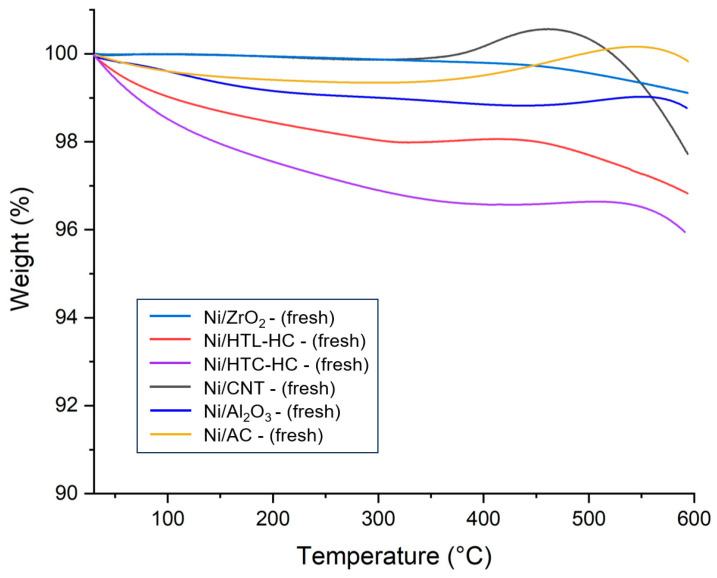
Thermogravimetric analysis (TGA) of analysis of fresh nickel catalysts supported on AC, CNT, HTC-HC, HTL-HC, Al_2_O_3_, and ZrO_2_ supports.

The catalytic stability is a key parameter for selecting a suitable catalyst for the SCWG reaction. To determine the stability of supported nickel catalysts, thermogravimetric analysis (TGA) of fresh supported nickel catalysts was performed. Figure 3 represents the TGA curves for all fresh catalysts. From Figure 3, it can be observed that all catalysts suffered a small amount of weight loss up to 200 °C. This can be attributed to the desorption and removal of any adsorbed gases and moisture from the environment. Interestingly, the weight of catalysts seems to start increasing at temperatures near 450–600 °C. This weight gain can be assigned to the oxidation of nickel metal present in the catalysts. Weight gain in Ni/ZrO_2_ was lowest in this range, indicating that the Ni/ZrO_2_ has strong metal–support interaction, preventing the oxidation of metallic nickel.

**Figure 4:**
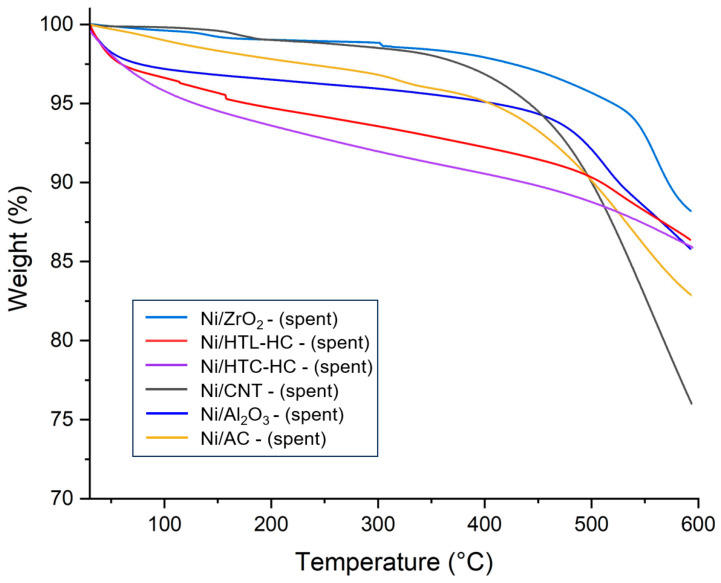
Thermogravimetric analysis (TGA) of spent nickel catalysts supported on AC, CNT, HTC-HC, HTL-HC, Al_2_O_3_, and ZrO_2_ supports.

In SCWG, catalyst deactivation could be attributed to active metal sintering, phase transformation of support, and coking. Deactivation of nickel catalysts occurs primarily due to coke deposition. Coke deposition tendency of catalysts reduces its activity in reaction and impacts its reusability. Catalysts with less coke deposition usually have better activity and stability in the reaction. The TGA of spent catalysts also represents the coke deposition on the surface of the catalyst. To evaluate the coke deposition behavior of Ni-based catalysts, TGA analysis of spent catalysts recovered from SCWG of canola straw was performed, and mass loss was observed. Figure 4 represents the TGA curves of spent catalysts used in the gasification of canola straw in SCW. From Figure 4, it can be seen that in all catalysts, a mass loss was majorly observed in two regions. In the first region, up to a temperature of 200 °C, weight loss could be assigned to the desorption of moisture on the catalytic surface [29]. In the second region, beyond 350 °C, major mass loss was ascribed to the oxidation of deposited coke on the catalysts’ surface [29]. In spent catalysts, the Ni/ZrO_2_ catalyst demonstrated the least amount of mass loss, followed by the Ni/Al_2_O_3_ catalyst. Interestingly, all carbon supports suffered a drastic mass loss beyond 550 °C except the Ni/CNT catalyst. This was due to the oxidation of carbon supports resulting in the loss of catalyst mass.

Results of catalytic gasification of canola straw with nickel catalysts supported on AC, CNT, HTL-HC, HTC-HC, ZrO_2_, and Al_2_O_3_ at 500 °C, 10 wt%, and 1 h are presented in Table 2. It can be identified that the Ni/Al_2_O_3_ catalyst showed the highest total gas yield of 35.3 mmol/g, followed by 33.0 mmol/g for Ni/ZrO_2_. The higher gas yield of Ni/Al_2_O_3_ can be attributed to its ability to enhance the reforming and hydrolysis of canola straw, which promoted the gasification of canola straw for the production of gaseous products. In SCWG, usually, for liquid feedstock, catalysts with higher surface areas show better catalytic activity as compared to catalysts with lower surface areas. However, despite having a high surface area and the highest pore volume, the gas yield obtained with the use of the Ni/CNT catalyst was the lowest at 27.1 mmol/g, which was even lower than the total gas yield obtained in the non-catalytic run. Similarly, the Ni/AC catalyst also had the highest surface area and very high pore volume, but it too performed poorly, with a total gas yield of 30.9 mmol/g. A possible reason for the poor performance of Ni/AC with a higher surface area compared to Ni/Al_2_O_3_ can be assigned to coke deposition identified via BET surface area, as well as lower Ni dispersion and metal sintering during the SCWG reaction, as indicated by XRD analysis. Among all the carbon-based nickel-supported catalysts, Ni/HTC-HC had the highest total gas yield of 32 mmol/g. This shows that even though higher surface area catalysts are beneficial for SCWG of lignocellulosic biomass, other factors such as metal dispersion and catalyst deactivation via coking and sintering play an important role in deciding the catalytic activity of catalysts during gasification in SCW.

**Figure 5:**
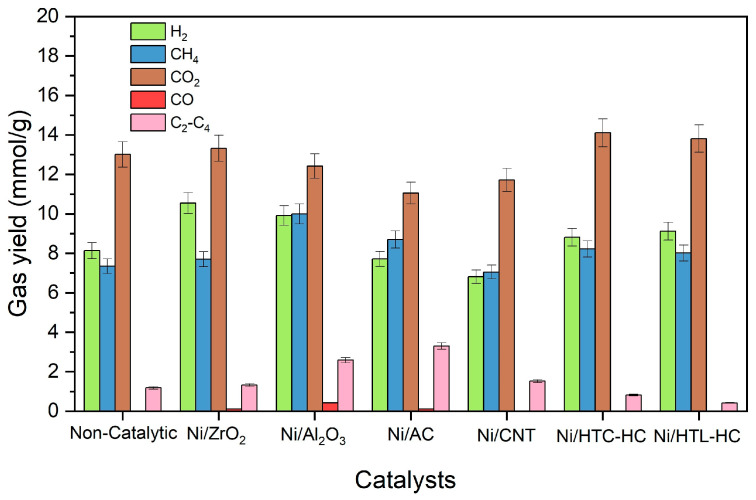
Results of individual gas yields of catalytic SCWG of canola straw with nickel catalysts supported on AC, CNT, HTC-HC, HTL-HC, Al_2_O_3_, and ZrO_2_ supports at reaction conditions of 500 °C, 60 min, 10 wt%, and 23–25 MPa.

Individual gas yields of SCWG of canola straw with supported nickel catalysts are presented in Figure 5. It can be observed that the Ni/ZrO_2_ catalyst showed the highest hydrogen yield of 10.5 mmol/g. This is due to the basic nature of Ni/ZrO_2_, which promotes reforming and water–gas shift reactions and enhances the hydrogen yield. Kou et al. [35] also observed an increase in hydrogen yield with the use of a Ni/ZrO_2_ catalyst for SCWG of oil-containing wastewater. They reported approximately 360% rise in hydrogen yield with the use of the Ni/ZrO_2_ catalyst compared to a non-catalytic run. They concluded that the Ni/ZrO_2_ favored the reforming and water–gas shift reaction, which facilitated the cleavage of C=C bonds, resulting in a high yield of hydrogen and gaseous products. Statistical significance of the results was analyzed using ANOVA to measure any statistically significant differences between the means of the gas yields. Unequal variance *t*-tests (Welch’s *t*-tests) were used to compare the difference in mean hydrogen yield from the catalytic run with those from the non-catalytic run. ANOVA analysis confirmed the difference in the means of all gas yields with very high significance (*p*-values < 0.05) and with high values of F-Statistics (25.5–365.9). Furthermore, a *t*-test showed a significant difference in hydrogen yield of catalysts compared to the non-catalytic run, with high significance. It also showed that the mean hydrogen yield using Ni/AC and Ni/CNT was lower compared to that from the non-catalytic run.

Interestingly, despite showing the highest total gas yield, the Ni/Al_2_O_3_ catalyst showed a relatively lower hydrogen yield. This might be due to the acidic nature of Ni/Al_2_O_3_ catalysts compared to Ni/ZrO_2_ [32]. The acidic nature of Ni/Al_2_O_3_ retards the water–gas shift reaction while enhancing the methanation reaction [22]. This limits the formation of hydrogen, and the produced hydrogen is also consumed for the production of methane via the methanation reaction. This was also made evident by the higher methane and CO yield and lower CO_2_ yield of the Ni/Al_2_O_3_ catalyst. Additionally, the Ni/ZrO_2_ catalyst was more stable compared to Ni/Al_2_O_3_, as demonstrated by the TGA analysis. The lowest hydrogen yield was demonstrated by Ni/CNT and Ni/AC catalysts with hydrogen yields of 6.8 mmol/g and 7.7 mmol/g, respectively. Poor hydrogen yield of Ni/CNT and Ni/AC catalysts can be attributed to diminishing reforming and WGS reactions while favoring other side reactions, such as methanation and hydrogenation reactions. These reactions increase methane yield while decreasing the hydrogen yield.

Overall, the Ni/ZrO_2_ catalyst demonstrated high activity for the gasification of canola straw in SCW, with the highest hydrogen yield and hydrogen selectivity. Due to its superior performance and stability among all supported catalysts, Ni/ZrO_2_ was further modified with the promotors and evaluated for SCWG of canola straw.

### 3.2. SCWG Gas Yields of Promoted Ni/ZrO_2_ Catalysts

Due to the promising results of Ni/ZrO_2_, it was modified with the addition of potassium (K), zinc (Zn), and cerium (Ce) promotor. Promotors are used to improve the hydrogen yield and selectivity while also providing increased stability to the catalyst. Potassium was selected because it is an alkali earth metal that enhances the WGS reaction to improve the hydrogen yield. Tavasoli et al. [33] used potassium as a promotor and witnessed its promoting effect in SCWG of sugarcane bagasse with Cu supported on Al_2_O_3_ catalysts. Zn promotors have demonstrated a high effectivity in decreasing methane and hydrogen-consuming reactions [36]. Zn blocks the adsorption of H_2_ and CO on the active sites of the catalyst [37]. For Ni-based catalysts, Ce proved to be an effective promotor in methane reforming processes [38] and for SCWG of glucose [39].

**Figure 6:**
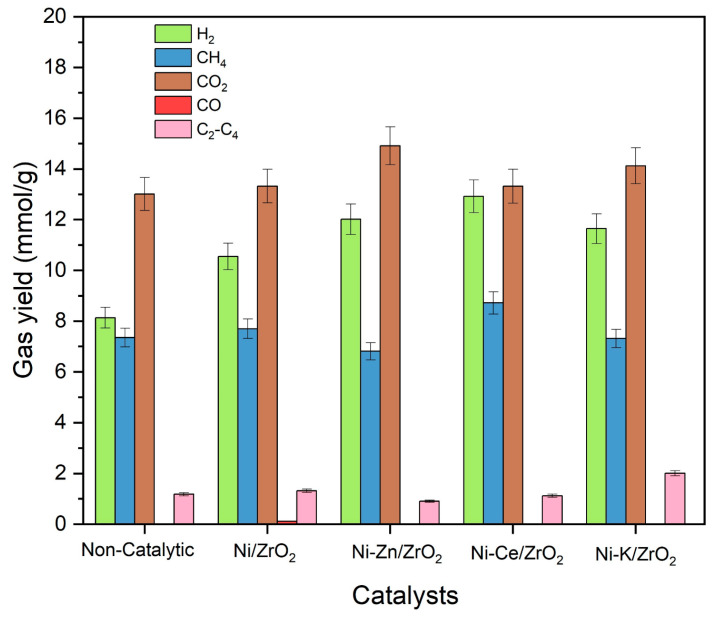
Results of individual gas yields of catalytic SCWG of canola straw with K, Zn, and Ce promoted ZrO_2_ catalysts at reaction conditions of 500 °C, 60 min, 10 wt%, and 23–25 MPa.

Promoted Ni/ZrO_2_ catalysts were utilized in catalytic SCWG of canola straw at reaction conditions of 500 °C, 10 wt%, 23–25 MPa, and 60 min to evaluate the comparative performance of the promotors for Ni/ZrO_2_ catalysts. Results of individual gas yield, total gas yield, H_2_ selectivity, and LHV are presented in Table 2 and Figure 6. It can be observed from Table 2 that the total gas yield improved from 29.7 in a non-catalytic run to 33.0 mmol/g with the addition of Ni/ZrO_2_. However, the addition of promotors significantly increased the total gas yield to 36.1 mmol/g with the use of Ni-Ce/ZrO_2_, followed by (35.1 mmol/g) Ni-K/ZrO_2_ and (34.7 mmol/g) Ni-Zn/ZrO_2_. The addition of Ni-Ce/ZrO_2_ also increased the LHV of gaseous products to 5243 kJ/Nm^3^, compared to 4761 kJ/Nm^3^ of the unpromoted Ni/ZrO_2_ catalyst and 4271 kJ/Nm^3^ of the non-catalytic run. Similarly, hydrogen yields in the non-catalytic run and with the unpromoted Ni/ZrO_2_ catalyst were 8.1 mmol/g and 10.5 mmol/g, respectively (Figure 6). Addition of promotors significantly further increased the hydrogen yield to 12.9 mmol/g with Ni-Ce/ZrO_2_, followed by 12.0 mmol/g with Ni-Zn/ZrO_2_ and 11.6 mmol/g with Ni-K/ZrO_2_ catalysts. ANOVA analysis confirmed the difference in the means of all gas yields for different promoted catalysts with high F-statistics (13.3–142.7) and high significance, and Welch’s *t*-tests showed an increase in the means of hydrogen yields with use of promotors compared to those using the unpromoted Ni/ZrO_2_ catalyst, with high significance.

Canola straw is a lignocellulosic biomass composed of complex structures of cellulose, hemicellulose, and lignin. The presence of lignin in biomass limits the degradation of biomass during gasification in SCW. Lignin also influences the decomposition of cellulose and hemicellulose, resulting in an overall lower yield of gaseous compounds. This results in lower total gas yield and subsequently lower hydrogen yield in non-catalytic runs. Since lignin has ester bonds joining its constituting phenyl propane molecules [40], this makes the hydrolysis of lignin in SCW difficult. The addition of promoted Ni-based catalysts enhances the hydrolysis and reforming of lignin by facilitating the cleavage of ester bonds and ring-opening reactions. This leads to improved overall gasification of lignocellulosic biomass, as well as higher total gas yield and hydrogen yield with the use of promoted nickel-based catalysts.

Figure 6 represents the individual gas yields of canola straw in the presence of promoted Ni-based catalysts. The nickel-based catalysts improved the yield of hydrogen, CO_2_ and CH_4_. The addition of an alkali metal promotor to the Ni/ZrO_2_ catalyst improved the hydrogen yield at the expense of the CO yield compared to the unpromoted Ni/ZrO_2_ catalysts. This is due to the catalytic action of alkali metal in promoting the water–gas shift reaction by forming a formate intermediate compound increasing the conversion of CO for the production of hydrogen. This limits the methanation of CO, which reduces methane yield, resulting in a higher yield of hydrogen and a lower yield of methane.

The addition of Ce promotors to the Ni/ZrO_2_ catalyst significantly enhanced the yield of hydrogen and CH_4_ gases while reducing the yield of CO compared to the unprompted Ni/ZrO_2_ catalytic run. High yields with Ni-Ce/ZrO_2_ can be assigned to its ability to promote the reforming reactions to enhance the gasification of canola straw in SCW. This also led to the highest LHV of 5243 kJ/Nm^3^ of gaseous products obtained with the use of Ce-promoted Ni/ZrO_2_ catalyst. Additionally, the Ce promotor facilitates the oxidation of the coke and char, which minimizes the coke deposition on the catalyst’s surface. This results in high stability and activity of Ce-promoted Ni/ZrO_2_ catalysts, resulting in a high yield of gaseous products.

Interestingly, the addition of Zn promotor enhanced the hydrogen and CO_2_ gas yields while showing the lowest CO yield. This is due to the enhanced reforming and water–gas shift reactions, which enhanced the yield of H_2_ and CO_2_ at the expense of CO. However, it resulted in the lowest yield of methane (6.8 mmol/g) among all runs, including unpromoted and non-catalytic runs. It also showed the lowest yield of C_2_-C_4_ hydrocarbons compared to all promoted and unpromoted Ni/ZrO_2_ catalytic runs. This can be explained by the fact that the addition of Zn inhibited the hydrogenation of CO to minimize the formation of methane and heavy hydrocarbons in the reaction. Zn forms a layer on the Ni surface, which restricts the adsorption of CO and H_2_ on its catalytic surface. This results in the diminution of methanation and hydrogenation reactions, while the rate of water–gas shift reaction remains unaffected. Thus, it results in an increased yield of H_2_ and CO_2_ while reducing the methane yield. This was further substantiated by its high hydrogen selectivity of 34.7%.

### 3.3. X-ray Diffraction Analysis of Modified Ni/ZrO_2_ Catalysts

**Figure 7:**
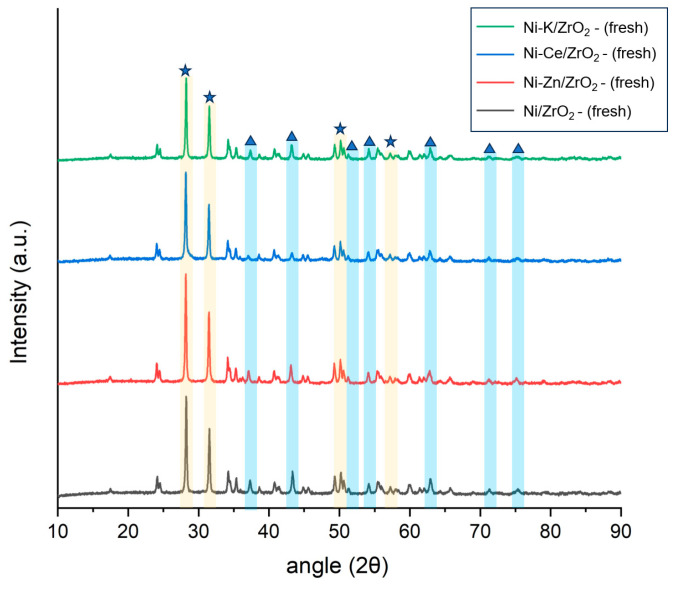
X-ray diffraction analysis (XRD) analysis of fresh K, Zn, and Ce promoted ZrO_2_ catalysts. NiO is represented as (▲) and ZrO_2_ is represented as (★) in the curve.

X-ray diffraction analysis (XRD) of freshly promoted Ni/ZrO_2_ catalysts was performed to investigate the crystallographic properties of these catalysts. Results of the XRD analysis are presented in Figure 7. It can be seen that all promoted Ni/ZrO_2_ catalysts show crystalline peaks of monoclinic ZrO_2_ at 2θ of 28°, 31°, 34°, and 56° and tetragonal ZrO_2_ peaks at 2θ of 30°, 35°, 51°, and 60°. The peak observed at 2θ of 37.5°, 44°, 61.5°, and 76° degrees represents the presence of the NiO. It can also be observed from Figure 7 that the addition of promotors decreased the intensity of the NiO peaks compared to the unpromoted Ni/ZrO_2_ catalyst. The extent of the decrement of the peak intensities of NiO was highest in Ni-Ce/ZrO_2_, followed by Ni-K/ZrO_2_ and Ni-Zn/ZrO_2_. The lowest intensity of NiO peaks with Ni-Ce/ZrO_2_ demonstrated the increased dispersion of NiO particles with the addition of Ce promotors. High dispersion of nickel species in Ni-Ce/ZrO_2_ improves the effective surface area of active metal, resulting in improved reaction kinetics of hydrolysis, reforming, and water–gas shift reactions to enhance hydrogen yield.

**Figure 8:**
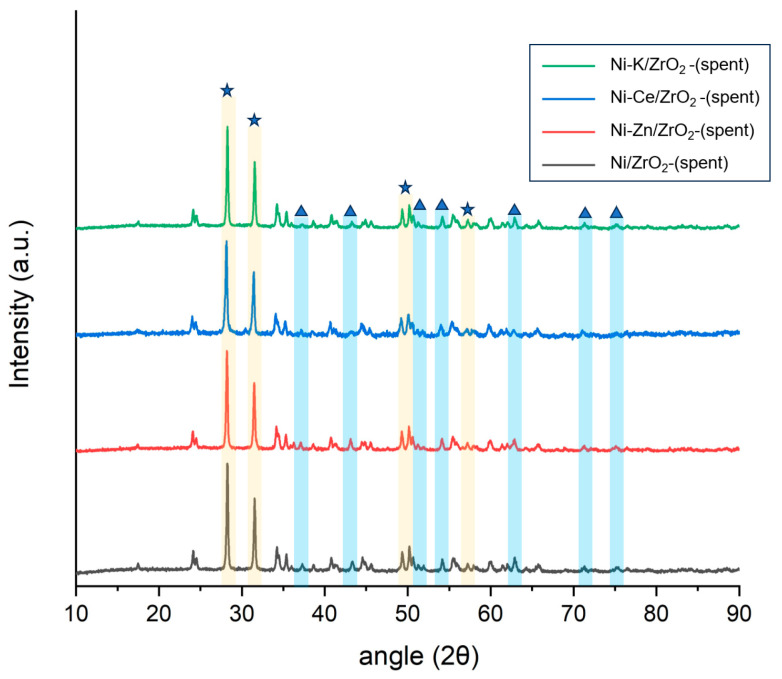
X-ray diffraction analysis (XRD) analysis of spent K, Zn, and Ce promoted ZrO_2_ catalysts. NiO is represented as (▲) and ZrO_2_ is represented as (★) in the curve.

XRD analysis of spent catalysts after being used in SCWG of canola straw showed that for all spent promoted Ni/ZrO_2_ catalysts, the relative intensity of monoclinic ZrO_2_ peaks at the 2θ angle of 28°, 31°, 34°, and 56° increased, while the intensities of tetragonal ZrO_2_ peaks at the 2θ angle of 30°, 35°, 51°, and 60° decreased compared to fresh catalysts (Figure 8). This is due to the transformation of the tetragonal phase of ZrO_2_ into the monoclinic ZrO_2_ phase during the SCWG reaction. Furthermore, the intensity of the NiO peaks in all spent catalysts increased compared to fresh catalysts. This is similar to results obtained from XRD analysis of unpromoted catalysts, which represents the sintering of Ni metal in spent catalysts. It should be noted that the increase in the intensities of NiO peaks was minimal in Ni-Ce/ZrO_2_, followed by Ni-Zn/ZrO_2_, Ni-K/ZrO_2_, and Ni/ZrO_2_. This shows that the Ce promotor prevented the sintering of the Ni-Ce/ZrO_2_ catalysts by restricting the growth of nickel particle size via agglomeration.

### 3.4. BET Analysis of Promoted Ni/ZrO_2_ Catalysts

Results of BET analysis of freshly promoted Ni/ZrO_2_ catalysts are shown in Table 3. It can be observed that the addition of promotors to Ni/ZrO_2_ catalysts decreased the surface area of the catalysts. The order of surface area of catalysts was (5.1 m^2^/g) Ni-K/ZrO_2_ > (4.4 m^2^/g) Ni-Zn/ZrO_2_ > (3.9 m^2^/g) Ni-Ce/ZrO_2_ compared to the BET surface area of 6.0 m^2^/g of unpromoted Ni/ZrO_2_ catalyst. Similarly, the addition of promotors also decreased the pore volume of the catalysts. This decrease in BET surface area and pore volume with the addition of promotors is due to the introduction of the metal particles of promotors in the catalyst pore. This blocks the pores and reduces the available surface area and pore volume of the catalysts. Su et al. [23] also made similar observations for promoted Ni catalysts. They reported a decrease in the pore volume and surface area of the Ni/Al_2_O_3_ catalysts with the introduction of the La promotors. They also observed that an increase in La promotor loading further decreases the pore volume and surface area of the catalyst.

Even though the addition of promotors resulted in a decrement in the surface area, it does not necessarily translate into the poor gasification of biomass in the SCW. Promotors prevent the active nickel particles from coagulating, which reduces the metal sintering by preventing the growth of nickel particles during the gasification of biomass in SCW. Additionally, due to the hydrogen splitting ability of the promotors, split hydrogen ions can spill over to nickel active metal to enhance the reduction in nickel particles and result in the sustained high performance of the catalysts during the reaction. This also prevents the deactivation of catalysts and improves the gas yields of the SCWG process.

Furthermore, the analysis of the spent promoted Ni/ZrO_2_ catalysts after use in SCWG of canola straw revealed that, similar to unpromoted catalysts, promoted catalysts also suffered a loss in surface area and pore volume. However, the extent of the reduction in surface area and pore volume in spent catalysts compared to fresh catalysts was low in promoted catalysts compared to unpromoted catalysts. This can be attributed to the textural stability provided by the promotors to catalysts, which limited the sintering of catalysts by restricting the growth of Ni particles during the gasification. The lowest change in surface area and pore volume in spent catalysts compared to fresh catalysts was observed in the Ce-promoted Ni/ZrO_2_ catalysts, followed by Zn- and K-promoted Ni/ZrO_2_ catalysts. This is due to the enhanced dispersion of nickel particles due to the addition of the Ce promotor, as identified by the XRD analysis of the Ni-Ce/ZrO_2_ catalyst. Additionally, Ce promotor could also oxidize the coke deposited on the catalyst. This reduces the blockage of the pores of the catalysts caused by coke and Ni particles, resulting in stable surface area and pore volume of the catalysts over their use in SCWG. It also explains the highest total gas yield and hydrogen yield obtained with the Ni-Ce/ZrO_2_ catalyst.

**Figure 9:**
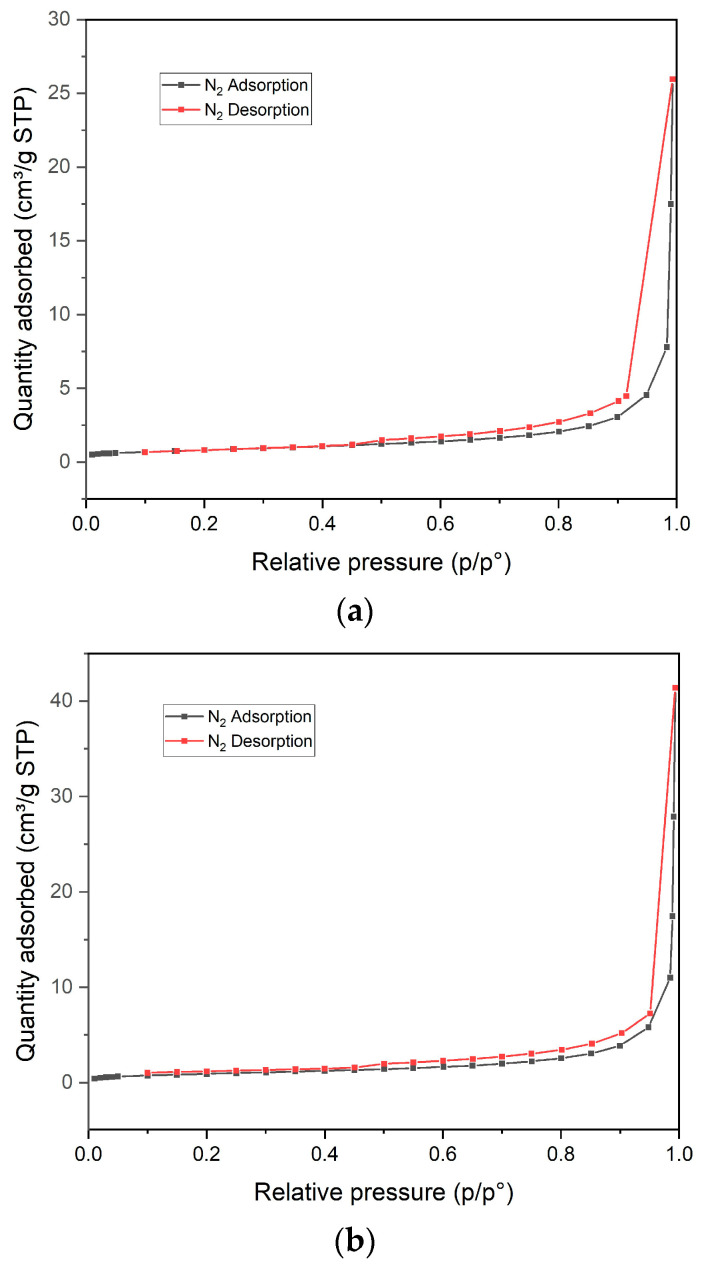
(**a**) Brunauer–Emmett–Teller (BET) adsorption and desorption curve of fresh Ni-Ce/ZrO_2_ catalyst. (**b**) Brunauer–Emmett–Teller (BET) adsorption and desorption curve of spent Ni-Ce/ZrO_2_ catalyst.

BET adsorption and desorption isotherms of fresh and spent Ni-Ce/ZrO_2_ catalysts revealed a type IV isotherm (Figure 9). According to the International Union of Pure and Applied Chemistry (IUPAC), type IV isotherm is indicative of the presence of mesopores, where the adsorption and desorption curves do not overlap, indicating the presence of capillary condensation [35]. It also showed the type H3 hysteresis loop, which does not exhibit any limiting adsorption at high values, and the lack of a plateau at high pressures suggests that the material does not have a uniform pore structure.

### 3.5. TGA Analysis of Promoted Ni/ZrO_2_ Catalysts

**Figure 10:**
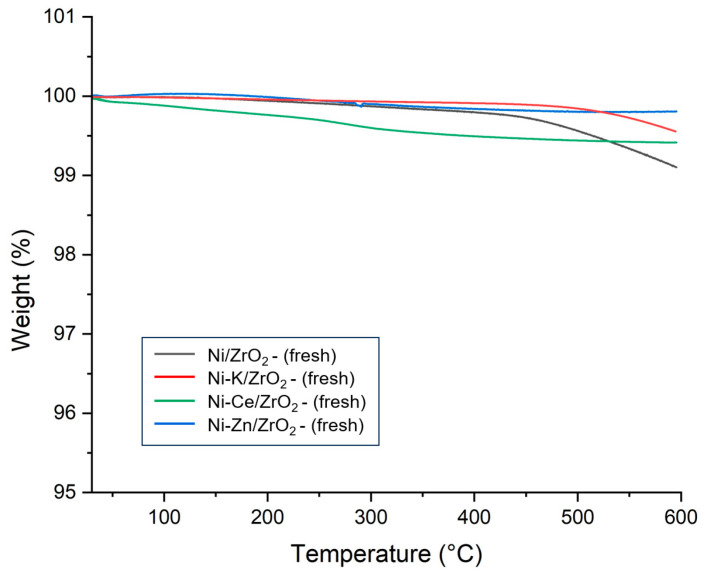
Thermogravimetric analysis (TGA) of fresh K, Zn, and Ce promoted ZrO_2_ catalysts.

The thermal stability of promoted Ni/ZrO_2_ catalysts was determined using the TGA, and results are presented in Figure 10. It can be observed that all promoted Ni/ZrO_2_ catalysts suffered a very minimum mass loss similar to unpromoted fresh catalysts. A very small weight loss was observed in the range of 200 °C due to the desorption of adsorbed gas and moisture. A very marginal hike in catalyst weight was noticed in the range of 450–600 °C due to the oxidation of nickel. However, total mass change was low in promoted Ni/ZrO_2_ catalysts compared to unpromoted Ni/ZrO_2_ catalysts. Minimum weight change was observed in the Ni-Ce/ZrO_2_ catalyst. This is due to the improved strength of interaction of nickel metal with support with the addition of Ce promotor, which prevented the oxidation of the catalyst and enhanced the thermal stability of the Ni-Ce/ZrO_2_ catalyst.

**Figure 11:**
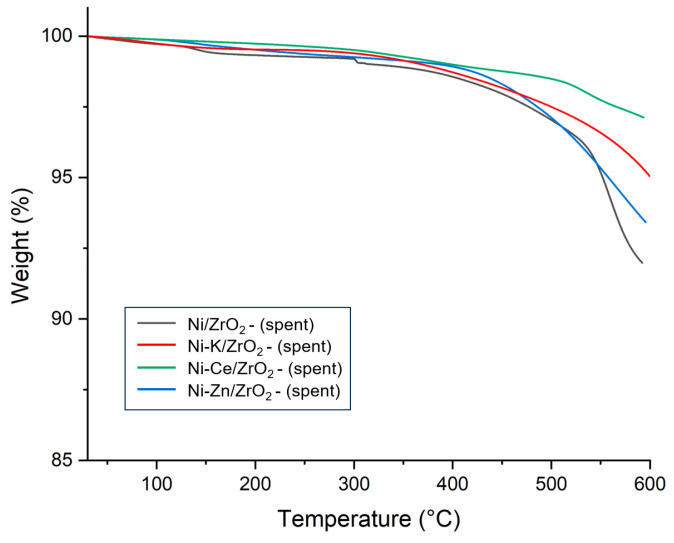
Thermogravimetric analysis (TGA) of spent K, Zn, and Ce promoted ZrO_2_ catalysts.

To analyze the ability of promoters to prevent coke deposition during the SCWG reaction, TGA of the spent catalysts of the SCWG reaction of canola straw was performed. Results of the TGA of the spent promoted catalysts are presented in Figure 11. In the TGA analysis of spent catalysts, two counter effects were taking place which influenced the change in the spent catalysts’ weight. First, catalysts suffered mass loss due to the oxidization of the coke deposited on the surface of the catalysts. However, the oxidation of active metal also increased the weight of the catalysts by forming a metal oxide on the surface of the catalysts. Oxidation of carbon occurred in two phases; at a low-temperature range, mostly oxidation of amorphous carbon which was easier to oxidize took place [41]. However, at higher temperature ranges, there were mostly graphitized carbon oxides, depending on their crystallization [42]. It can be identified that the addition of the promotors reduced the percentage of mass loss of the catalysts compared to the 10% mass loss observed for unpromoted Ni/ZrO_2_ catalysts. This represents the fact that the addition of the promotor reduced the carbon deposition on the catalyst and improved the stability of the catalyst in the reaction.

The Ce-promoted Ni-Ce/ZrO_2_ catalyst demonstrated the lowest mass loss of 3%, followed by the 5% mass loss of the Ni-K/ZrO_2_ and the 7% mass loss of the Ni-Zn/ZrO_2_ catalyst. This shows that the coke deposition in Ni-Ce/ZrO_2_ was minimal, which can be attributed to strong metal–promotor interactions and higher dispersion of the nickel in the Ni-Ce/ZrO_2_ catalyst. The ability of Ce promotors to prevent the deactivation of Ni-Ce/ZrO_2_ catalysts from coke deposition contributed to their having the highest hydrogen and total gas yield among all catalysts. Thus, the ability to restrict the coke formation via a catalyst is an important parameter and strongly influences the performance of the catalyst in the SCWG of biomass. Kang et al. [28] also observed that the addition of Ce promotor to Ni supported by Al_2_O_3_ catalyst for SCWG of lignin decreased the coke deposition and enhanced the gas yields. The addition of Ce promotor improved the hydrogen yield by nearly 175% and the total gas yield by approximately 57% compared to unpromoted catalysts.

The high stability of Ni-Ce/ZrO_2_ against coke formation and its high activity during gasification in SCW are due to the redox ability of the Ce promotor. Ce has two stable oxidation states of Ce^3+^ and Ce^4+^ [43]. This gives Ce the ability to store and release oxygen via redox shift between its two oxidation states [44]. It allows Ce to rapidly mobilize oxygen over the catalyst via its oxygen uptake and release it in a reversible redox reaction [43,45].

Ce^4+^ ⇌ Ce^3+^ + O*_l_*

This lattice oxygen produced at the Ce surface can partially oxidize solid coke adsorbed on the surface of the catalyst to form CO [46].

C + O*_l_* → CO + O_*l*−1_

O_*l*−1_ results in the formation of the reduced site on the surface of Ce.

Lattice oxygen can also react with produced CO on the catalyst’s surface for oxidation of CO into CO_2_. Increased formation of CO_2_ instead of CO can restrict the coke deposition.

CO + O*_l_* → CO_2_ + O_*l*−1_

This imparts high coke resistance to the Ni-Ce/ZrO_2_ catalyst and enhances the stability of the catalyst. Furthermore, nickel oxide present in Ni-Ce/ZrO_2_ catalyst can also react with the lattice oxygen of Ce to be reduced in the metallic form. It increases the dispersion of the nickel metal and increases active sites in the Ni-Ce/ZrO_2_ catalyst. In the case of Ni-K/ZrO_2_-promoted catalyst, alkali metals are known to increase the alkalinity of the catalysts and enable CO_2_ chemisorption [47]. This increases the number of oxygen vacancies and improves the dispersion of Ni metal, whereas for the Ni-Zn/ZrO_2_ catalyst, the Zn promotor also increases the alkalinity of the catalysts and has strong synergetic effects with nickel metal, which imparts high thermal stability with high metal dispersion to the catalyst [48]. To further assess the morphology of the Ni-Ce/ZrO_2_ catalyst, SEM analysis of Ni-Ce/ZrO_2_ catalyst was performed.

### 3.6. SEM and ICP Analysis of Promoted Ni/ZrO_2_ Catalysts

**Figure 12:**
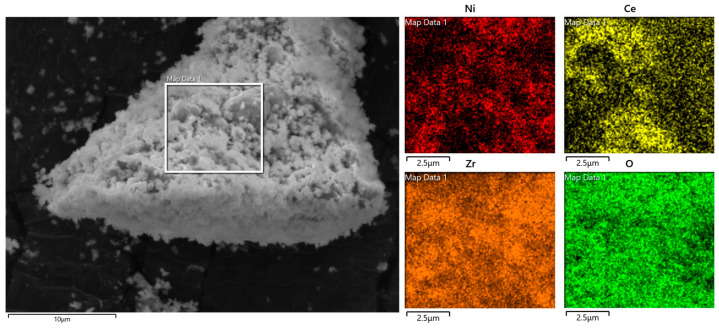
Scanning electron microscopy (SEM) images and elemental mapping of fresh Ni-Ce/ZrO_2_ catalyst.

Surface morphology and the dispersion of elements of catalysts were analyzed using scanning electron microscopy–energy dispersive X-ray analysis (SEM-EDX). Elemental analysis of catalysts was also performed using ICP-OES analysis. Figure 12 shows the SEM and elemental mapping of the fresh Ni-Ce/ZrO_2_ catalyst. It can be observed from Figure 12 that fresh samples have a cluster-like structure. It also highlights the irregular shape of the particles and the variation in their particle sizes. Elemental mapping using EDX shows uniform and fine dispersion of active metals and promotors on the catalyst surface. This confirms the uniform distribution and loading of precursors in the incipient wetness impregnation and co-impregnation methods. It also shows the presence of promotors, active metals, and supports for the promoted Ni-Ce/ZrO_2_ catalyst. Elemental analysis of catalysts in bulk using ICP-OES also showed the presence of precursors with a 9.9 ± 0.4 wt% amount of nickel on ZrO_2_. It also confirmed the appropriate precursor loading on the catalysts.

**Figure 13:**
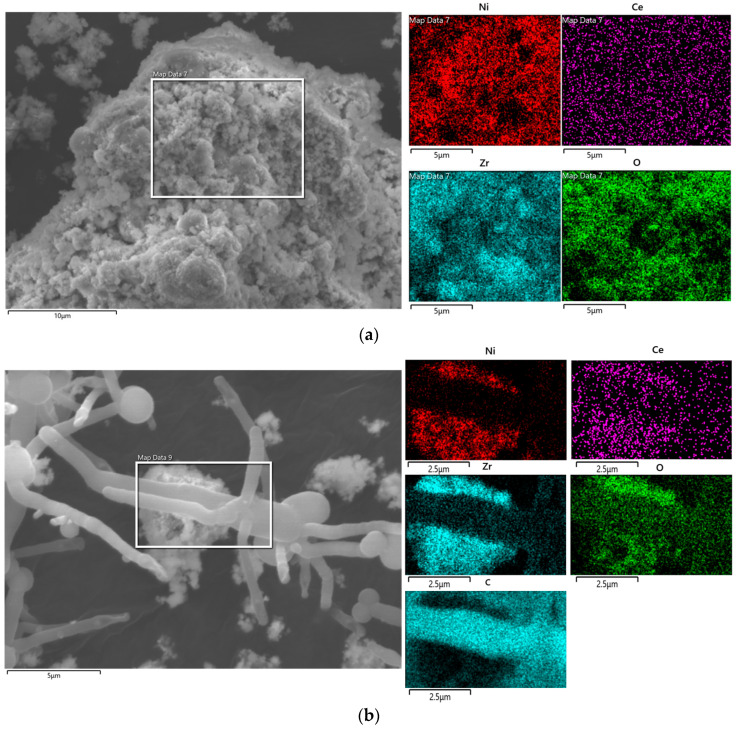
(**a**) Scanning electron microscopy (SEM) images and elemental mapping of spent Ni-Ce/ZrO_2_ catalyst. (**b**) Scanning electron microscopy (SEM) images and elemental mapping of fresh Ni-Ce/ZrO_2_ catalyst at higher magnification.

SEM-EDX and ICP analyses of spent catalysts were also performed to determine the morphological and elemental changes in catalysts after being used for SCWG of canola straw, and results are presented in Figure 13. From Figure 13a, it can be observed from the SEM analysis of spent catalysts that particles are larger due to agglomeration as compared to those of fresh catalysts, and the cluster-like shape observed in fresh catalysts was more compacted in spent catalysts, resulting in collapse of its pore structure after SCWG of canola straw. This highlights the sintering of the catalyst in SCWG reaction. Elemental mapping of used catalyst also showed variation compared to fresh catalyst. Elemental mapping showed the agglomeration of the active metal into larger crystals, and uniform dispersion of fresh catalysts became non-uniform and uneven after being used in SCWG reaction. These results are in agreement with XRD and BET analysis confirming the increase in the size of active metal crystals and the particle size of catalysts after the SCWG reaction. A higher-magnification SEM image of the spent catalysts in Figure 13b shows that the presence of carbon leads to formation of a long tail-like structure on the active metal surface of the spent catalyst, as identified by elemental mapping. This is due to the accumulation of coke generated via cracking during the SCWG reaction of canola straw. This is also in agreement with the TGA analysis highlighting the coking of catalysts in the SCWG reaction. ICP analysis of spent catalysts in bulk showed a similar composition compared to fresh catalysts, with 9.7 ± 0.5 wt% amount of nickel on ZrO_2_. This shows minimum change in the amount of precursor and the stability of the metal support framework during the SCWG of canola straw.

Thus, the Ni-Ce/ZrO_2_ catalyst suffered from sintering and coking during the SCWG reaction. However, the extent of catalyst deactivation of the Ni-Ce/ZrO_2_ catalyst was significantly lower than for the other catalysts. This shows the potential for reuse of the Ni-Ce/ZrO_2_ catalyst for multiple experimental runs of SCWG of canola straw. Thus, the reusability of Ni-Ce/ZrO_2_ catalyst was compared with that of an unpromoted Ni/ZrO_2_ catalyst to examine the effectiveness of the Ce promotor in improving the reusability and stability of promoted Ni-Ce/ZrO_2_ catalyst compared to unpromoted Ni/ZrO_2_ catalyst.

### 3.7. Reusability of Ni-Ce/ZrO_2_ Catalysts

Owing to the ability to recover the heterogeneous catalysts after the completion of the reaction, the reusability of the catalysts plays an important role in the selection of the suitable catalysts. The reusability of catalysts not only reduces the amount of fresh catalyst required but also improves the economics of the process. The reusability of Ni-Ce/ZrO_2_ catalyst over its repeated use for gasification of canola straw in SCW was tested to evaluate its catalytic stability. It was then compared with the reusability of Ni/ZrO_2_ catalyst to evaluate the effect of Ce promotor on minimizing the deactivation of the Ni-Ce/ZrO_2_ catalyst. Catalysts were recovered from the SCWG reactor after the completion of the SCWG reaction. Two catalytic re-runs were performed without regeneration to test coke deposition and sintering of catalysts. All the catalytic runs were performed at 500 °C, 23–25 MPa, 10 wt%, and 60 min.

**Figure 14:**
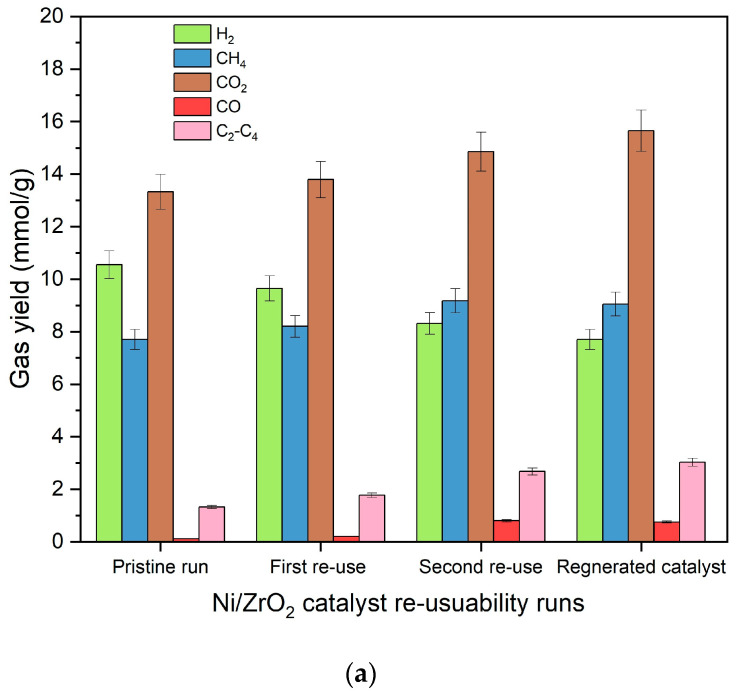

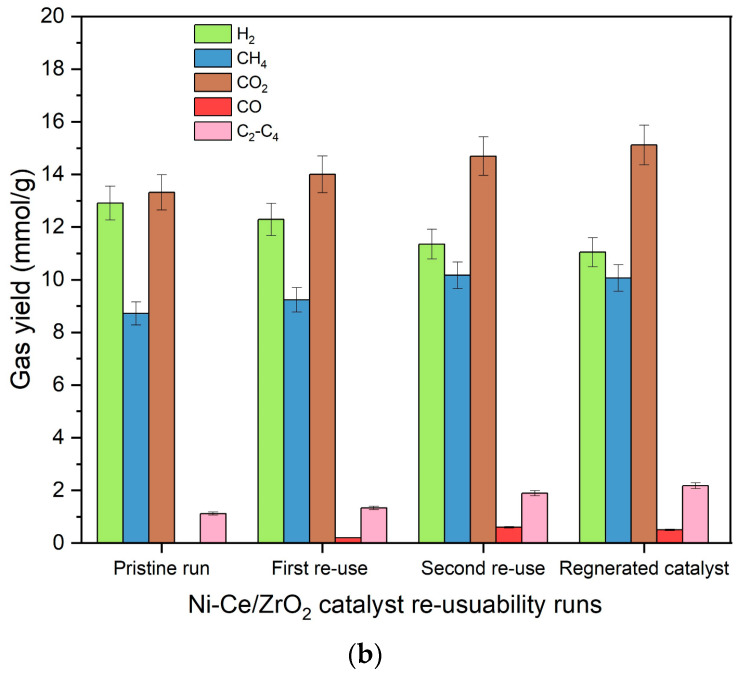
(**a**) Results for individual gas yields of reusability of ZrO_2_ catalyst over its repeated use for gasification of canola straw in supercritical water at reaction conditions of 500 °C, 60 min, 10 wt%, and 23–25 MPa. (**b**) Results for individual gas yields of reusability of Ni-Ce/ZrO_2_ catalyst over its repeated use for gasification of canola straw in supercritical water at reaction conditions of 500 °C, 60 min, 10 wt%, and 23–25 MPa.

Results for the reusability of Ni/ZrO_2_ and Ni-Ce/ZrO_2_ catalysts are presented in Figure 14. It can be observed from Figure 14 that reusing both Ni/ZrO_2_ and Ni-Ce/ZrO_2_ catalysts after the first run caused them to suffer decrements in the hydrogen yield. For Ni/ZrO_2_, hydrogen yield decreased to 9.6 mmol/g in the first reuse run, compared to the yield of 10.5 mmol/g for pristine Ni/ZrO_2_ catalysts. Ni-Ce/ZrO_2_ catalysts also suffered a decrement in hydrogen yield to 12.3 mmol/g in the first reuse from 12.9 mmol/g from the pristine Ni-Ce/ZrO_2_ catalytic run. A similar trend was observed for the second reuse of the Ni/ZrO_2_ catalyst, in which hydrogen yield further decreased to 8.3 mmol/g. For the Ni-Ce/ZrO_2_ catalyst, too, hydrogen yield further decreased to 11.4 mmol/g in the second reuse of the Ni-Ce/ZrO_2_ catalyst. Ni/ZrO_2_ catalyst suffered a loss of 8% in the first reuse cycle and 21% in the second reuse cycle compared to the pristine Ni/ZrO_2_ catalyst run for gasification of canola straw in SCW. Even though the Ni-Ce/ZrO_2_ catalyst also suffered a loss in hydrogen yield, the addition of the Ce promotor reduced the extent of hydrogen yield in its reuse cycles. Only 5% and 12% decrements were observed in hydrogen yield for the first reuse and second reuse cycle, respectively, of the Ni-Ce/ZrO_2_ catalyst, compared to the pristine Ni-Ce/ZrO_2_ catalytic run. This shows the superior thermal stability of the Ni-Ce/ZrO_2_ catalyst compared to the unpromoted Ni/ZrO_2_ catalyst. Su et al. [23] also observed a similar decrement in hydrogen yield for La-promoted Ni/Al_2_O_3_ catalysts for gasification of food waste. They reported an 87% drop in hydrogen production in the third run cycle for unpromoted Ni/Al_2_O_3_ catalysts. This drop in hydrogen yield was minimized by the addition of La promotor, and a 65% drop in hydrogen production was observed in the third run cycle for the La-promoted Ni/Al_2_O_3_ catalyst.

On the contrary, the yield of methane and C_2_-C_4_ hydrocarbons increased from 8.7 and 1.1 mmol/g in the pristine Ni-Ce/ZrO_2_ catalytic run to 9.2 and 1.3 mmol/g in the first reuse of the catalyst. Similarly, the yield of methane and C_2_-C_4_ hydrocarbons further increased to 10.2 and 1.9 mmol/g in the second reuse of the Ni-Ce/ZrO_2_ catalyst. Ni/ZrO_2_ catalyst also observed a similar rise in yield of methane and C_2_-C_4_ hydrocarbons over its reuse cycles. This is due to the enhancement of methanation and secondary reactions over reforming and water–gas shift reactions during the reuse of catalysts, which is due to the reduced activity of catalysts over their repeated use. This enhanced the yield of methane and heavy molecular hydrocarbon gases while decreasing the hydrogen yield. Interestingly, despite the diminution of reforming and water–gas shift reactions, yields of CO and CO_2_ witnessed a continuous rise over repeated use of the catalysts. However, this rise in yield of CO and CO_2_ is due to the oxidation of the coke deposited on the surface of the catalysts.

A major reason for decrement in the catalytic activity was due to the coke deposition and active metal sintering. Loss of activity of catalysts due to coke deposition is reversible where regeneration of catalysts in the presence of oxygen will restore the catalytic activity of the catalyst. However, metal sintering is an irreversible process that results in permanent loss of catalytic activity. To test the cause of the loss of activity of the catalysts, catalysts were also regenerated by performing calcination and reduction of the recovered used catalysts after the second rerun, and then they were utilized to conduct the gasification of canola straw in SCW at 500 °C, 23–25 MPa, 10 wt%, and 60 min. Results of the activity of regenerated catalysts for gasification of canola straw in SCW are also presented in Figure 14. From Figure 14, it can be observed that the regeneration of catalysts did not improve catalytic activity and still resulted in a decrement to 11.0 mmol/g in hydrogen yield, compared to 11.4 mmol/g for the second reuse run and 12.9 mmol/g for the pristine Ni-Ce/ZrO_2_ catalytic run. This shows that the loss of the catalytic activity of Ni-Ce/ZrO_2_ is primarily due to sintering of nickel metal. Statistical analysis using ANOVA also confirmed the differences in the means of all gas yields for reused and regenerated catalysts for both Ni-Ce/ZrO_2_ and Ni/ZrO_2_ catalysts, with high F-statistics ((11.7–553.1) and (17.58–730.2), respectively), and with high significance. Welch’s *t*-tests also confirmed a decrease in the mean of hydrogen yield in reused and regenerated catalysts compared to that from the pristine run for both Ni-Ce/ZrO_2_ and Ni/ZrO_2_ catalysts.

Overall, the addition of Ce promotor to Ni/ZrO_2_ also enhanced the reusability of the Ni-Ce/ZrO_2_ catalyst. Comparison of performance of the Ni-Ce/ZrO_2_ catalyst with reported modified nickel-based catalysts for SCWG of lignocellulosic biomass also demonstrated its superior catalytic activity for SCWG (Table 4). Therefore, due to high catalytic activity, thermal stability, reusability, metal dispersion, and lower sintering and coking of Ni-Ce/ZrO_2_ catalyst, the addition of Ce promotor successfully improved the performance of the Ni/ZrO_2_ catalyst. Ni-Ce/ZrO_2_ proved to be the most suitable catalyst for the gasification of canola straw in SCW.

## 4. Conclusions

In this study, an in-depth analysis was conducted for novel nickel-based catalysts, and six supports (AC, CNT, HTC-HC, HTL-HC, Al_2_O_3_, ZrO_2_) were systematically screened, followed by screening of three promotors (K, Zn, and Ce) for the most suitable promoted and supported nickel catalyst. The catalytic activity of catalysts was tested for gasification of canola straw in supercritical water at 500 °C, 23–25 MPa, 10 wt%, and 60 min. Among all supported catalysts, Ni/ZrO_2_ demonstrated superior catalytic activity for SCWG of canola straw, having the highest hydrogen yield at 10.5 mmol/g compared to 8.1 mmol/g for the non-catalytic run. Ni/ZrO_2_ favored the reforming and water–gas shift reactions, which enhanced the hydrogen yield. XRD and TGA analysis revealed that the superior performance of Ni/ZrO_2_ was due to its high metal dispersion, strong metal–support interaction, and thermal stability. Despite having the highest BET surface area at 590 m^2^/g, Ni/CNT performed the worst among all the catalysts, having the lowest hydrogen yield of 6.8 mmol/g. This confirms that even though a high surface area of the catalyst is desirable, for SCWG, active metal dispersion, active metal crystalline phase, and metal–support interaction play a pivotal role in catalytic activity. Interestingly, among nickel catalysts supported on carbon based supports, nickel catalysts supported on hydrochars obtained from HTL and HTC performed better than Ni/AC and Ni/CNT, with hydrogen yields of 9.1 and 8.8 mmol/g, respectively.

Due to the superior catalytic activity of Ni/ZrO_2_, it was further modified with the addition of three promotors (K, Ce, and Zn). The addition of the promotor enhanced the hydrogen yield, and the order of hydrogen yield for the promoted catalyst was (12.9 mmol/g) Ni-Ce/ZrO_2_ > (12.0 mmol/g) Ni-Zn/ZrO_2_ and (11.6 mmol/g) Ni-K/ZrO_2_ catalysts, compared to 10.5 mmol/g yield with the unpromoted Ni/ZrO_2_ catalyst. The superior performance of the Ce promotor was due to its ability to improve the metal dispersion, thermal stability, reducibility, and metal–support interaction while minimizing the metal sintering, as confirmed by the XRD and TGA analysis of the catalysts. The Ce promotor also had an oxygen uptake and release ability, improving the mobility of oxygen and reducing the coke deposition on the catalyst for superior thermal stability of the Ni-Ce/ZrO_2_ catalyst. SEM-EDX and ICP analysis confirmed the optimum loading of the active metal and promotor on support and their uniform dispersion on support. SEM-EDX analysis of spent catalysts also confirmed the formation of coke and agglomeration of active species, as identified by TGA, BET, and XRD analysis.

The reusability of the best-performing Ni-Ce/ZrO_2_ catalyst was determined by the stability of the catalyst over its repeated use. Recycling the Ni-Ce/ZrO_2_ catalyst after one run dropped its hydrogen yield to only 5% and reusing it after two runs only caused a 12% drop in its hydrogen yield compared to the pristine Ni-Ce/ZrO_2_ catalyst. This confirmed the superior reusability of Ni-Ce/ZrO_2_ catalyst over repeated uses for SCWG of canola straw. Regeneration of the catalyst after its multiple uses and reusing it for gasification of canola straw in SCW showed that the deactivation of the catalyst was primarily due to the sintering of active metal.

Even though use of the Ce promotor in the Ni-Ce/ZrO_2_ catalyst significantly improved the performance, activity, selectivity, and stability of the catalysts for production of hydrogen from the SCWG of canola straw, the modified Ni-Ce/ZrO_2_ catalyst suffered from higher amounts of production of other gases, such as CH_4_ and CO_2_. This resulted in low hydrogen selectivity and low hydrogen yield. Furthermore, use of Ni-Ce/ZrO_2_ catalyst still did not yield 100% gasification of canola straw, which shows that room can be made for a higher amount of hydrogen yield by further modifying this catalyst. Thus, more research is needed on more effective promotors and bimetallic catalysts aiming to enhance the reforming and water–gas shift reactions to maximize the hydrogen gas yield via gasification of canola straw. Additionally, more sustainable and biobased catalysts can be explored to improve the environmental impact of the catalytic SCWG process. Nevertheless, this study demonstrated the potential of Ni-Ce/ZrO_2_ as a catalyst for SCWG of lignocellulosic biomass.

## Figures and Tables

**Table 1:** Results of BET analysis of fresh and spent nickel catalysts supported on AC, CNT, HTC-HC, HTL-HC, Al_2_O_3_, and ZrO_2_ supports for SCWG of canola straw.

		Ni/Al_2_O_3_	Ni/AC	Ni/CNT	Ni/HTL-HC	Ni/HTC-HC	Ni/ZrO_2_
	Surface area (m^2^/g)	280	590	291	59	45	6
Fresh	Total pore volume (cm^3^/g)	0.58	0.68	0.93	0.07	0.03	0.02
	Pore size (nm)	8.9	6.8	17.1	12.6	14.2	12.8
	Surface area (m^2^/g)	92	158	81	42	33	4.82
Spent	Total pore volume (cm^3^/g)	0.44	0.34	0.54	0.05	0.02	0.18
	Pore size (nm)	9.1	8.5	20.3	13.8	16.4	13.2

**Table 2:** Results of total gas yield, lower heating value (LHV), and hydrogen selectivity of catalytic SCWG of canola straw at reaction conditions of 500 °C, 60 min, 10 wt%, and 23–25 MPa.

Catalyst		Total Gas Yield (mmol/g)	H_2_ Selectivity (%)	LHV (kJ/Nm^3^)
Screening of Supports	Non-catalytic	29.7	27.4	4271
	Ni/ZrO_2_	33.0	32.0	4761
	Ni/Al_2_O_3_	35.3	28.1	6363
	Ni/AC	30.9	25.0	6072
	Ni/CNT	27.1	25.1	4237
	Ni/HTC-HC	32.0	27.6	4427
	Ni/HTL-HC	31.4	29.1	4128
Screening of Promotors	Ni/ZrO_2_	33.0	32.0	4761
	Ni-Zn/ZrO_2_	34.7	34.7	4323
	Ni-Ce/ZrO_2_	36.1	35.8	5243
	Ni-K/ZrO_2_	35.1	33.2	5160
Reusability of Ni/ZrO_2_ Catalyst	Pristine run	33.0	32.0	4761
	First re-use	33.6	28.7	5145
	Second re-use	35.8	23.2	5996
	Regenerated	36.2	21.3	6103
Reusability of Ni-Ce/ZrO_2_ Catalyst	Pristine run	36.1	35.8	5243
	First re-use	37.1	33.2	5519
	Second re-use	38.7	29.3	6160
	Regenerated	38.9	28.4	6254

**Table 3:** Results of BET analysis of fresh and spent K, Zn, and Ce promoted ZrO_2_ catalysts for SCWG of canola straw.

		Ni/ZrO_2_	Ni-K/ZrO_2_	Ni-Zn/ZrO_2_	Ni-Ce/ZrO_2_
	Surface area (m^2^/g)	6.0	5.1	4.4	3.9
Fresh	Total pore volume (cm^3^/g)	0.02	0.19	0.02	0.02
	pore size (nm)	12.8	12.1	12.7	15.6
	Surface area (m^2^/g)	4.82	4.61	4.29	3.75
Spent	Total pore volume (cm^3^/g)	0.18	0.18	0.02	0.02
	pore size (nm)	13.2	12.9	13.4	15.9

**Table 4:** Summary of studies of nickel-based catalytic SCWG of lignocellulosic biomass.

Feedstock	Catalyst	Process Conditions	H_2_ Yield	References
Wheat straw	Ni/MgO	Temperature = 450 °C, Pressure = 23–28 MPa, Time = 20 min, Feed concentration = 7.4 wt%	11.6 mmol/g	[22]
Food waste	Ni-La/Al_2_O_3_	Temperature = 480 °C, Pressure = 26–30 MPa, Time = 40 min, Feed concentration = 8 wt%	8.03 mmol/g	[23]
Timothy grass, wheat straw, canola meal	Ni-Ce/Al_2_O_3_	Temperature = 650 °C, Pressure = 26 MPa, Time = 50 min, Feed concentration = 16.67 wt%	1.9 mmol/g (Canola meal), 2.01 mmol/g (wheat straw), 1.54 (timothy grass)	[49]
Glucose	Ni-Mg/Al_2_O_3_	Temperature = 400 °C, Pressure = 22.1 MPa, Time = 20 min, Feed concentration = 5 wt%	11.8 mmol/g	[50]
Food waste	Ni/Al_2_O_3_	Temperature = 360 °C, Pressure = 20 MPa, Time = 90 min, Feed concentration = 10 wt%	1.88 mmol/g	[51]
Canola straw	Ni-Ce/ZrO_2_	Temperature = 500 °C, Pressure = 23–25 MPa, Time = 60 min, Feed concentration = 10 wt%	12.9 mmol/g	This study

## Data Availability

Data available upon request.

## Abbreviations

AAEMs	Alkali and alkaline earth metals
AC	Activated carbon
GHGs	Greenhouse gases
HC	Hydrochar
HTC	Hydrothermal carbonization
HTC-HC	Hydrothermal liquefaction-hydrochar
HTL	Hydrothermal liquefaction
HTL-HC	Hydrothermal carbonization-hydrochar
Mt	Million tons
WGS	Water–gas shift

## References

[1] Wright V.P. (2023). World Energy Outlook.

[2] Energy Production and Consumption—Our World in Data. https://ourworldindata.org/energy-production-consumption.

[3] Climate Change the Greatest Threat the World Has Ever Faced, UN Expert Warns | OHCHR. https://www.ohchr.org/en/press-releases/2022/10/climate-change-greatest-threat-world-has-ever-faced-un-expert-warns.

[4] Ahad M.T., Bhuiyan M.M.H., Sakib A.N., Becerril Corral A., Siddique Z. (2023). An Overview of Challenges for the Future of Hydrogen. Materials.

[5] Nunes R.F., Costa D., Ferraria A.M., Botelho do Rego A.M., Ribeiro F., Martins Â., Fernandes A. (2023). Heterogenization of Heteropolyacid with Metal-Based Alumina Supports for the Guaiacol Gas-Phase Hydrodeoxygenation. Molecules.

[6] Attia M., Farag S., Chaouki J. (2020). Upgrading of Oils from Biomass and Waste: Catalytic Hydrodeoxygenation. Catalysts.

[7] Bampaou M., Panopoulos K., Seferlis P., Voutetakis S., Matino I., Petrucciani A., Zaccara A., Colla V., Dettori S., Annunziata Branca T. (2021). Integration of Renewable Hydrogen Production in Steelworks Off-Gases for the Synthesis of Methanol and Methane. Energies.

[8] IEA (2022). Global Hydrogen Review 2022.

[9] Pascual A.R., Víctor E.E., Martín C., Broda M., Yelle D.J., Serwá Nska K. (2022). Bioethanol Production from Lignocellulosic Biomass—Challenges and Solutions. Molecules.

[10] Canola Industry in Canada, from Farm to Global Markets. https://www.canolacouncil.org/about-canola/industry/#:~:text=Canola%20production%26text=Canola%20is%20grown%20by%2043%2C000,of%20all%20farm%20crop%20receipts.

[11] Acharya B., Dutta A. (2016). Fuel Property Enhancement of Lignocellulosic and Nonlignocellulosic Biomass through Torrefaction. Biomass Convers. Biorefinery.

[12] Marcuello C., Foulon L., Chabbert B., Aguié-Béghin V., Molinari M. (2020). Atomic Force Microscopy Reveals How Relative Humidity Impacts the Young’s Modulus of Lignocellulosic Polymers and Their Adhesion with Cellulose Nanocrystals at the Nanoscale. Int. J. Biol. Macromol..

[13] Lewandowski W.M., Ryms M., Kosakowski W. (2020). Thermal Biomass Conversion: A Review. Processes.

[14] Dutzi J., Boukis N., Sauer J. (2023). Process Effluent Recycling in the Supercritical Water Gasification of Dry Biomass. Processes.

[15] Rodriguez Correa C., Kruse A. (2018). Supercritical Water Gasification of Biomass for Hydrogen Production—Review. J. Supercrit. Fluids.

[16] Khandelwal K., Nanda S., Boahene P., Dalai A.K. (2023). Hydrogen Production from Supercritical Water Gasification of Canola Residues. Int. J. Hydrogen Energy.

[17] Qu X., Zhou X., Yan X., Zhang R., Bi J. (2018). Behavior of Alkaline-Metal Catalysts in Supercritical Water Gasification of Lignite. Chem. Eng. Technol..

[18] Feng H., Zhou Z., Hantoko D., Zhong L., Ar Rahim D., Fang W., Yan M. (2021). Effect of Alkali Additives on Desulfurization of Syngas during Supercritical Water Gasification of Sewage Sludge. Waste Manag..

[19] García-Jarana M.B., Portela J.R., Sánchez-Oneto J., Martinez de la Ossa E.J., Al-Duri B. (2020). Analysis of the Supercritical Water Gasification of Cellulose in a Continuous System Using Short Residence Times. Appl. Sci..

[20] Heeley K., Orozco R.L., Macaskie L.E., Love J., Al-Duri B. (2023). Supercritical Water Gasification of Microalgal Biomass for Hydrogen Production—A Review. Int. J. Hydrogen Energy.

[21] Demey H., Ratel G., Lacaze B., Delattre O., Haarlemmer G., Roubaud A. (2023). Hydrogen Production by Catalytic Supercritical Water Gasification of Black Liquor-Based Wastewater. Energies.

[22] Lu Y., Jin H., Zhang R. (2019). Evaluation of Stability and Catalytic Activity of Ni Catalysts for Hydrogen Production by Biomass Gasification in Supercritical Water. Carbon Resour. Convers..

[23] Su H., Kanchanatip E., Wang D., Zhang H., Antoni, Mubeen I., Huang Z., Yan M. (2020). Catalytic Gasification of Food Waste in Supercritical Water over La Promoted Ni/Al_2_O_3_ Catalysts for Enhancing H_2_ Production. Int. J. Hydrogen Energy.

[24] Khandelwal K., Dalai A.K. (2023). Integration of Hydrothermal Gasification with Biorefinery Processes for Efficient Production of Biofuels and Biochemicals. Int. J. Hydrogen Energy.

[25] Khoshbouy R., Takahashi F., Yoshikawa K. (2019). Preparation of High Surface Area Sludge-Based Activated Hydrochar via Hydrothermal Carbonization and Application in the Removal of Basic Dye. Environ. Res..

[26] da Silva Alves D.C., Healy B., Pinto L.A.d.A., Cadaval T.R.S., Breslin C.B. (2021). Recent Developments in Chitosan-Based Adsorbents for the Removal of Pollutants from Aqueous Environments. Molecules.

[27] Isaeva V.I., Vedenyapina M.D., Kurmysheva A.Y., Weichgrebe D., Nair R.R., Nguyen N.P.T., Kustov L.M. (2021). Modern Carbon–Based Materials for Adsorptive Removal of Organic and Inorganic Pollutants from Water and Wastewater. Molecules.

[28] Kang K., Azargohar R., Dalai A.K., Wang H. (2016). Systematic Screening and Modification of Ni Based Catalysts for Hydrogen Generation from Supercritical Water Gasification of Lignin. Chem. Eng. J..

[29] Hossain M.Z., Chowdhury M.B.I., Charpentier P.A. (2019). Effect of Surface Acidity of Al_2_O_3_ Supported Metal Catalysts on Catalytic Activity and Carbon Deposition during SCWG of Glucose. Biomass Bioenergy.

[30] Zhu B., Li S., Wang W., Zhang H. (2019). Supercritical Water Synthesized Ni/ZrO_2_ Catalyst for Hydrogen Production from Supercritical Water Gasification of Glycerol. Int. J. Hydrogen Energy.

[31] Lv P.M., Xiong Z.H., Chang J., Wu C.Z., Chen Y., Zhu J.X. (2004). An Experimental Study on Biomass Air–Steam Gasification in a Fluidized Bed. Bioresour. Technol..

[32] Neill J.L., Wang S., Bergin E.A., Rista Lestari A., Mariyah Ulfa S. (2019). Effect of Support on the Hydrodeoxygenation of Phenol Using Ni-Based Metal Catalysts: Ni/SiO_2_, Ni/ZrO_2_, and Ni/Al_2_O_3_. IOP Conf. Ser. Mater. Sci. Eng..

[33] Tavasoli A., Barati M., Karimi A. (2016). Sugarcane Bagasse Supercritical Water Gasification in Presence of Potassium Promoted Copper Nano-Catalysts Supported on γ-Al_2_O_3_. Int. J. Hydrogen Energy.

[34] Li S., Guo L., Zhu C., Lu Y. (2013). Co-Precipitated Ni–Mg–Al Catalysts for Hydrogen Production by Supercritical Water Gasification of Glucose. Int. J. Hydrogen Energy.

[35] Kou J., Xu J., Jin H., Guo L., Zhang D., Cao W. (2018). Evaluation of Modified Ni/ZrO_2_ Catalysts for Hydrogen Production by Supercritical Water Gasification of Oil-Containing Wastewater. Int. J. Hydrogen Energy.

[36] Barati M., Babatabar M., Tavasoli A., Dalai A.K., Das U. (2014). Hydrogen Production via Supercritical Water Gasification of Bagasse Using Unpromoted and Zinc Promoted Ru/γ-Al_2_O_3_ Nanocatalysts. Fuel Process. Technol..

[37] Tamtögl A., Kratzer M., Killman J., Winkler A. (2008). Adsorption/Desorption of H_2_ and CO on Zn-Modified Pd(111). J. Chem. Phys..

[38] Yang X., Da J., Yu H., Wang H. (2016). Characterization and Performance Evaluation of Ni-Based Catalysts with Ce Promoter for Methane and Hydrocarbons Steam Reforming Process. Fuel.

[39] Lu Y., Li S., Guo L. (2013). Hydrogen Production by Supercritical Water Gasification of Glucose with Ni/CeO_2_/Al_2_O_3_: Effect of Ce Loading. Fuel.

[40] Kaur R., Goyal M. (2020). Isolation, Derivatization and Bioactive Properties of Natural Lignin Based Hydroxycinnamic Acids: A Review. Mini-Rev. Org. Chem..

[41] Kibis L.S., Svintsitskiy D.A., Stadnichenko A.I., Slavinskaya E.M., Romanenko A.V., Fedorova E.A., Stonkus O.A., Svetlichnyi V.A., Fakhrutdinova E.D., Vorokhta M. (2021). In Situ Probing of Pt/TiO_2_ Activity in Low-Temperature Ammonia Oxidation. Catal. Sci. Technol..

[42] Slavinskaya E.M., Kardash T.Y., Stonkus O.A., Gulyaev R.V., Lapin I.N., Svetlichnyi V.A., Boronin A.I. (2016). Metal–Support Interaction in Pd/CeO_2_ Model Catalysts for CO Oxidation: From Pulsed Laser-Ablated Nanoparticles to Highly Active State of the Catalyst. Catal. Sci. Technol..

[43] Wu Z., Jin R., Liu Y., Wang H. (2008). Ceria Modified MnOx/TiO_2_ as a Superior Catalyst for NO Reduction with NH3 at Low-Temperature. Catal. Commun..

[44] Bermejo M.D., Cocero M.J. (2006). Supercritical Water Oxidation: A Technical Review. AIChE J..

[45] Malyukin Y., Klochkov V., Maksimchuk P., Seminko V., Spivak N. (2017). Oscillations of Cerium Oxidation State Driven by Oxygen Diffusion in Colloidal Nanoceria (CeO_2−X_). Nanoscale Res. Lett..

[46] Kumar P., Sun Y., Idem R.O. (2008). Comparative Study of Ni-Based Mixed Oxide Catalyst for Carbon Dioxide Reforming of Methane. Energy Fuels.

[47] Tsiotsias A.I., Charisiou N.D., Yentekakis I.V., Goula M.A. (2020). The Role of Alkali and Alkaline Earth Metals in the CO_2_ Methanation Reaction and the Combined Capture and Methanation of CO_2_. Catalysts.

[48] Chatla A., Abu-Rub F., Prakash A.V., Ibrahim G., Elbashir N.O. (2022). Highly Stable and Coke-Resistant Zn-Modified Ni-Mg-Al Hydrotalcite Derived Catalyst for Dry Reforming of Methane: Synergistic Effect of Ni and Zn. Fuel.

[49] Kang K., Azargohar R., Dalai A.K., Wang H. (2016). Hydrogen Production from Lignin, Cellulose and Waste Biomass via Supercritical Water Gasification: Catalyst Activity and Process Optimization Study. Energy Convers. Manag..

[50] Li S., Guo L. (2019). Stability and Activity of a Co-Precipitated Mg Promoted Ni/Al_2_O_3_ Catalyst for Supercritical Water Gasification of Biomass. Int. J. Hydrogen Energy.

[51] Su H., Hantoko D., Yan M., Cai Y., Kanchanatip E., Liu J., Zhou X., Zhang S. (2019). Evaluation of Catalytic Subcritical Water Gasification of Food Waste for Hydrogen Production: Effect of Process Conditions and Different Types of Catalyst Loading. Int. J. Hydrogen Energy.

